# Characterization of bony changes localized to the cervical articular processes in a mixed population of horses

**DOI:** 10.1371/journal.pone.0222989

**Published:** 2019-09-26

**Authors:** Kevin K. Haussler, Roy R. Pool, Hilary M. Clayton

**Affiliations:** 1 Gail Holmes Equine Orthopaedic Research Center, Department of Clinical Sciences, College of Veterinary Medicine and Biomedical Sciences, Colorado State University, Fort Collins, Colorado, United States of America; 2 Texas A&M University, Department of Veterinary Pathobiology, Veterinary Medicine and Biomedical Sciences, College Station, Texas, United States of America; 3 Department of Large Animal Clinical Sciences, Michigan State University, East Lansing, Michigan, United States of America; Universita degli Studi di Perugia, ITALY

## Abstract

The objectives of this observational, cross-sectional study were to characterize and establish the prevalence of osseous proliferation of articular surfaces, joint margins and adjacent soft tissue attachments (i.e., joint capsule and deep spinal muscles) in a mixed population of horses of variable ages, sizes, and breeds to better capture the full spectrum of disease affecting the cervical articular processes. Cranial and caudal articular processes of the cervical and first three thoracic vertebrae (C2-T3) from 55 horses without a primary complaint of neck pain were evaluated for the presence and severity of abnormal bony changes. Data were analyzed to compare alterations in joint margin quadrants, paired articular surfaces within a synovial articulation, left-right laterality, and vertebral level distributions and to determine associations with age, wither height and sex. Seventy-two percent of articular processes had bony changes that were considered abnormal. Osteophyte formation was the most common bony change noted. Overall grades of severity included: normal (28%), mild (45%), moderate (22%), and severe (5%). The highest prevalence of mild changes was localized to the C3-C6 vertebral levels; moderate changes to C6-T2; and severe changes to C2-C3 and C6-T2. Most paired articular surfaces and left-right grades of severity were not significantly different. The grade of osseous pathology was positively associated with both age and wither height. A high prevalence and wide variety of abnormal bony changes of varying severity were found in articular processes across all vertebral levels. The clinical significance of the described lesions is unknown, but the findings are expected to enhance the reporting of articular process and periarticular changes noted on advanced diagnostic imaging of the equine cervical and cranial thoracic vertebral regions.

## Introduction

Neck pain and stiffness are becoming more commonly recognized clinical entities in horses due to increased awareness of potential adverse effects on athletic performance, use of detailed spinal evaluation procedures (e.g., acupuncture, chiropractic), and improved diagnostic imaging (e.g., ultrasonography, computed tomography) of this body region [1, 2]. Review of the equine literature suggests that cervical vertebral myelopathy is one of the primary disease processes affecting the equine cervical region [3, 4]. Concurrent osteophyte production has also been reported in studies on cervical vertebral instability; however, enlarged or encroaching articular processes associated with osteoarthritis are often considered a secondary contributing factor to spinal cord compression rather than as a primary disease process [5–7]. The clinical effects of cervical osteoarthritis in the absence of cervical myelopathy are not commonly reported or considered [8]. A further complication is that radiographic and histologic evidence of osteoarthritis is prevalent in horses with and without clinical signs of neck pain or stiffness [9, 10]. Therefore, little is known about the etiopathogenesis and clinical effects of osteoarthritis within the equine cervical vertebrae [11–13].

In humans, vertebral osteoarthritis is a clinical and pathological disorder that involves both structural and functional failure of the synovial articulations [14]. Osteoarthritis is often viewed as a disease of articular cartilage loss and bony proliferation; however, the process of failure involves the whole joint complex, which includes the cartilage, subchondral bone, synovium, joint capsule, ligaments, and periarticular paraspinal muscles and associated soft tissues. The spinal motion segment is considered the functional unit of the vertebral column, which includes the two dorsal synovial articulations and the ventrally-located intervertebral disk. These articular structures form a triad between adjacent vertebrae that support intervertebral motion and play an important role in load transmission [15]. Therefore, pathologic changes or failure of one of these constructs is expected to negatively affect the other two components, which may cloud the clinician’s ability to clearly isolate specific disease processes affecting the cervical articular processes, intervertebral disk or vertebral canal [16].

Osseous proliferation of the articular processes is reported to cause a wide variety of clinical syndromes in horses depending on the presence of local, peripheral or central effects. Locally, direct mechanical and inflammatory effects on cervical motion can produce signs of neck pain, muscle atrophy, stiffness in a specific direction, resistance to induced joint motion (e.g., baited stretches), and pain on palpation [3, 13]. Abnormal head and neck carriage may also occur if the upper cervical vertebrae are affected [17]. Peripherally, osseous proliferation has been reported to mechanically compress or chemically irritate the cervical nerve roots exiting the intervertebral foramen, which can produce signs of neck pain, local sweating, muscle atrophy, reduced performance, stumbling, and obscure forelimb lameness [12, 18, 19]. Centrally, changes in the articular process size, shape, and spatial orientation are commonly reported to change the internal contours of the vertebral canal and contribute to cervical myelopathy, which often produces signs of ataxia, paresis and spasticity that are typically more pronounced in the pelvic limbs, compared to the thoracic limbs [10, 20–22].

There are often discrepancies between the observed clinical signs and radiographic evidence of cervical osteoarthritis, which may be due to lack of specificity in clinical examinations or technological limitations for diagnostic imaging of this body region. Cervical radiography is complicated by the complex regional anatomy, superimposition of symmetric structures, and difficulty in obtaining orthogonal radiographic views [23]. Radiographic signs of cervical osteoarthritis involve both degenerative and proliferative features, which can include osteophyte formation, sclerosis and subchondral cysts within the articular process [12, 24, 25]. The technique of obtaining oblique view radiographs has improved diagnostic capabilities by revealing planar views of the cervical articular surfaces [9]. Ultrasonography of the cervical articular processes has also helped to identify joint effusion, capsular fibrosis, and periarticular bone proliferation (e.g., osteophytes) associated with osteoarthritis [26]. Advancements in computed tomography and magnetic resonance imaging of the equine neck have provided additional insights into the three-dimensional aspects of the cervical and cranial thoracic vertebrae and articular process modeling (e.g., changes in overall size, shape or angulation) [10]. Despite these advancements there continues to be a lack of specificity of imaging axial skeleton disorders.

With improved imaging modalities and increased interest in this body region, there is a need to fully describe the spectrum of disease within the cervical region to provide a common understanding of characteristic features of soft tissue and osseous pathology and to identify normal anatomical variants [27, 28]. Osteochondrosis and osteoarthritis are the two primary bone disorders that affect the cervical articular processes in horses [10, 29]. Osteoarthritic findings include capsular thickening, nodular erosions, enlargement of the joint surfaces, osteophyte formation, fragmentation, and ankylosis [30]. Interestingly, there are no reports in the equine literature of enthesophytes related to ossification of vertebral joint capsules or soft tissue attachments on or near the cervical articular processes. Topographically, osteoarthritis is not distributed evenly throughout the vertebral column [14]. Osteoarthritic changes are most commonly localized to the 5th, 6th and 7th cervical vertebrae and can occur in both young and old horses [30, 31]. With a current resurgence of training horses in extreme head and neck positions in both English and Western disciplines there is risk for induced joint disease throughout the entire cervicothoracic region (i.e., occiput to T3).

The objectives of the study were to characterize and establish the prevalence and vertebral distribution of bony changes localized to the cervical and cranial thoracic articular processes and to characterize the nature of those changes in a mixed population of horses (i.e., variable ages, sizes, breeds) to better capture the full spectrum of disease within this region and to localize osseous changes to specific articular and periarticular locations. Age and wither height relationships to increasing severity of bony changes were also assessed. We hypothesized that older, larger horses had proportionately more severe and widespread bony changes.

## Materials and methods

This observational, cross-sectional study was based on a necropsy survey assessing the prevalence of osseous pathology affecting paired articular processes from the cervical and first three thoracic vertebrae (caudal C2 to caudal T3) of 55 mixed breed horses obtained from Michigan State University, Animal Diagnostic Center. The horses included in the study represented a diverse population of breeds, which included miniature horses, ponies, Arabian, Morgan, Missouri Foxtrotter, Quarter Horses, Paint, Appaloosa, Thoroughbred, Warmbloods, Draft horses, and crossbreds. The average age of horses was 13 ± 7 years. The age categories consisted of young (1–7 years of age, N = 13); mature (between 8–15 years, N = 20) and old (>15 years of age, N = 22) horses. Horses within the wither height categories included small (<1.45 m; N = 6); medium (1.45–1.70 m; N = 42) and large (>1.75 m; N = 7). The sex distribution consisted of 35 males and 20 females.

The first three thoracic vertebrae were included due to increased awareness of the clinical relevance of disease in the cervicothoracic region in performance horses. This was a convenience sample consisting of horses that were presented for euthanasia or that died or were euthanized in the veterinary teaching hospital. None of the horses in the study had a known primary complaint of neck pain or cervical-related disorders (e.g., ataxia). The horses were delivered dead (e.g., due to colic and for disposal) to the Animal Diagnostic Center or were euthanized in the clinic for reasons other than neck pain or were donated to this project due to old age or an incurable disease that was not neck-related. The subjects included in the study represented a diverse population of breeds, which included racing and non-racing horses and ponies.

Subjects were categorized according to age since a previous study indicated a higher prevalence and greater severity of osseous lesions of the thoracic and lumbar articular processes in older horses [32]. The age categories were based on general impressions of clinical relevance to osteoarthritis development and consisted of young (1–7 years of age); mature (between 8–15 years) and old (>15 years of age) subjects. Horses were also subdivided according to wither height, which is reported to be a good proxy for body mass [33]. The wither height categories included small (miniature horses and ponies; <1.45 m); medium (predominately Quarter Horses and Thoroughbreds; 1.45–1.70 m) and large (predominately Warmbloods and draft breeds; >1.75 m) horses.

**Fig 1:**
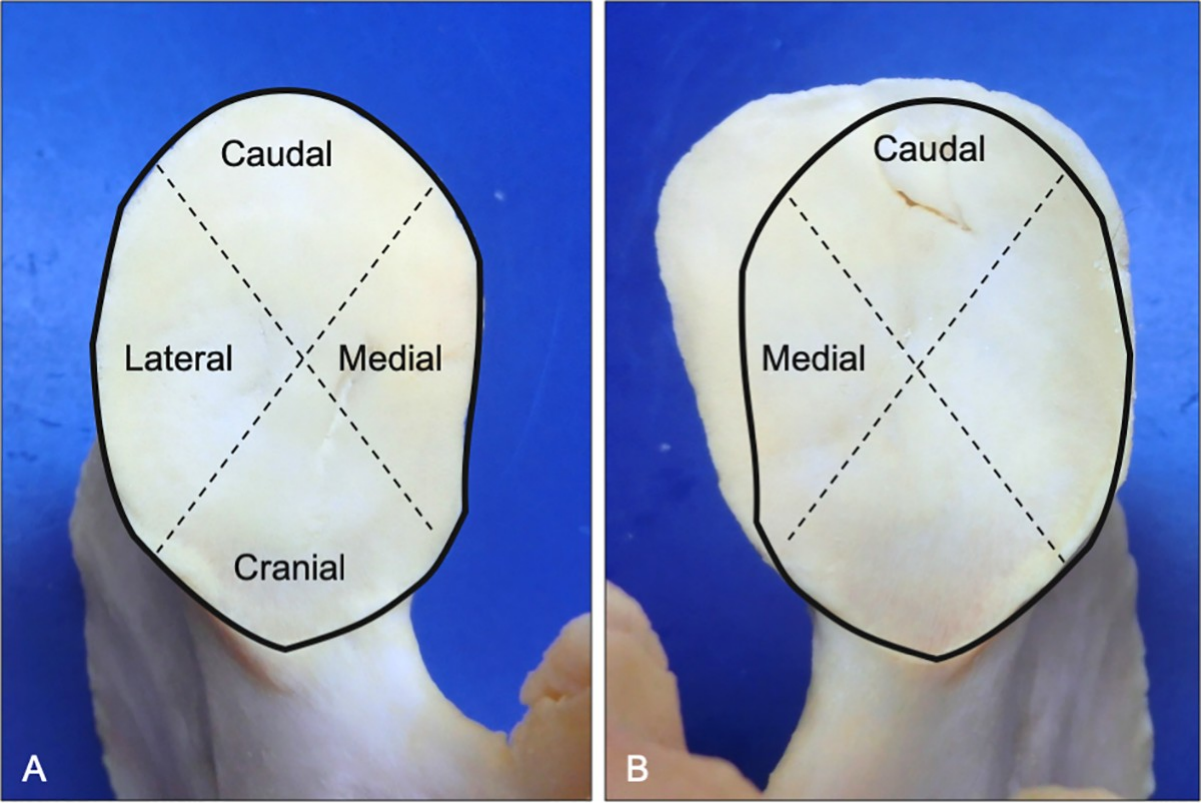
Topographical map used to define articular joint margin quadrants. The mirrored outline of the left articular surface (A) was overlaid the paired right caudal articular process (B). Note the size and shape asymmetry of the right articular surface that was localized to the caudomedial joint margins (B) of C2 from a 5-year-old Quarter Horse.

After euthanasia, the entire vertebral column and pelvis were removed intact and had all soft tissues removed by manual dissection and boiling of the vertebrae to allow full visualization of the cervical articular and periarticular osseous features. The cranial and caudal articular processes of the cervical and cranial thoracic vertebrae were evaluated for the presence of abnormal bony proliferation and changes in the size, shape and angulation of the articular surfaces. Normal cervical articular process morphology was characterized by 1) parallel articular surfaces, 2) thin, linear and smooth joint margins, and 3) being devoid of periarticular osteophytes and enthesophytes. Normal articular surfaces are typically ovoid in shape and have left-to-right symmetry in size, shape and angulation within a vertebral level [20]. Abnormal osseous proliferation was identified at articular joint margins (i.e., osteophytes) and to attachment sites of joint capsules and periarticular muscles (i.e., enthesophytes). For further characterization of the osseous changes, the anatomical locations of the affected joint margins were mapped to cranial, caudal, medial, and lateral quadrants (Fig 1), or described as periarticular if > 75% of the joint margin was affected.

Within each individual vertebra, the cranial-caudal and left-right articular processes were assessed and scored separately using the following system: Grade 0 = Normal, absent osseous changes; Grade 1 = mild, osseous changes affecting <25% of the articular or periarticular surfaces; Grade 2 = moderate, osseous changes affecting 25–50% of the articular or periarticular surfaces; and Grade 3 = severe, osseous changes affecting >50% of the articular or periarticular surfaces or the presence of enlarged vascular channels associated with retained epiphyseal vasculature. If enlarged vascular channels were present, it was assumed to represent a disease process with increased clinical relevance based on reports of similar changes within other skeletal regions [34, 35] and therefore, was scored as Grade 3 severity.

Individual articular processes were grossly evaluated for abnormal osseous proliferation, remodeling and the presence of enthesis. Articular process asymmetry was defined as left-right differences in size, shape, or angulation of the articular surfaces (Fig 1). Lipping was characterized as a pathologic overgrowth of bone along a joint margin and begins as a thin, sharp, bony projection that is contiguous with the articular joint margin. Flattening was characterized by an angular deviation and apparent depression of a joint margin relative to the normal planar articular surface. Modeling of the articular joint margins was defined as a grossly thickened, angular bony projection arising from the joint margin that extended from and was contiguous with the planar articular surface. Osteophytes were characterized as irregular bony proliferations located along the periarticular joint margins. Osteophytes are localized within the confines of the joint capsule and typically involve concurrent irregular bone surfaces and gross thickening due to cycling modeling of paired articular processes. In contrast, articular process thickening (without evidence of osteophytes) was defined as having an increased distance between the normal appearing joint margin and joint capsule insertion with smooth periarticular bone surfaces. Enlarged vascular channels were characterized by single to multifocal cavitations located between the articular joint margin and the site of joint capsule insertion. Ankylosis was defined as osseous proliferation that produced partial or complete bridging of opposing articular processes.

Periosteal callus was characterized as the presence of diffuse woven bone formation located near joint capsule or muscle insertion sites. Joint capsule entheses were characterized as mature bony proliferation at the site of articular process joint capsule insertions. Multifidi muscle entheses defined as osseous proliferation at the site of attachment along the dorsal aspect of the caudal articular processes. Intertransverse muscle entheses were characterized as osseous proliferation at the site of attachment along the ventral surface of cranial articular processes.

### Statistical analysis

The prevalence of all types, grades and anatomical locations of osseous changes were reported. The severity of bony changes within age, sex and wither height categories were assessed using Spearman’s rank-order correlation (p<0.05). Wilcoxon signed-rank statistical comparisons (p < 0.05) were used to assess cranial-caudal and left-right differences in osseous changes within individual synovial articulations or cervical vertebrae. Due to similar biological mechanisms (e.g., synovitis) affecting the cranial-caudal articular processes within a synovial articulation, we hypothesized that there would be no significant differences in the severity of osseous changes within paired articular processes. Similarly, we hypothesized that there would be no significant left-right differences in osseous changes within a cervical vertebra due to common biomechanical forces (e.g., head and neck carriage) that would be expected to produce bilateral bone or soft tissue microtrauma. To assess the presence of any “domino-effect” of osseous pathology migrating from one intervertebral joint to adjacent intervertebral articulations, we also compared unilateral osseous changes between cranial and caudal articular processes within individual vertebrae (e.g., left cranial and caudal articular processes of C3).

## Results

### Types of bony changes

Bony changes characterized as osteophytes or enthesophytes were identified in 72% of cervical and cranial thoracic (C2-T3) articular processes (n = 1,854) across all subjects. Variable degrees of severity of bony changes were observed with osteophytes noted to be the most common type of lesion (Table 1).

**Table 1:** Prevalence and associated grades of severity of abnormal osseous changes.

Osseous Changes	Prevalence	Mild	Moderate	Severe
Osteophytes	24% (n = 442)	12% (n = 231)	9% (n = 174)	2.0% (n = 37)
Flattening	24% (n = 437)	16% (n = 305)	7% (n = 130)	0.1% (n = 2)
Lipping	23% (n = 435)	21% (n = 390)	2% (n = 44)	0.1% (n = 1)
Modeling	11% (n = 204)	8% (n = 140)	3% (n = 58)	0.3% (n = 6)
Joint capsule enthesis	6% (n = 105)	3% (n = 47)	3% (n = 51)	0.4% (n = 7)
Extension impingement	4% (n = 83)	2% (n = 33)	2% (n = 45)	0.3% (n = 5)
Thickening	3% (n = 59)	2% (n = 33)	1% (n = 25)	0.1% (n = 1)
Enlarged vascular channels	3% (n = 47)	1% (n = 20)	1% (n = 22)	0.3% (n = 5)
Muscle enthesis	2% (n = 44)	1% (n = 16)	1% (n = 24)	0.2% (n = 4)
Asymmetry	1% (n = 18)	1% (n = 16)	0.1% (n = 1)	0.1% (n = 1)
Periosteal callus	1% (n = 11)	0.2% (n = 3)	0.3% (n = 6)	0.1% (n = 2)
Ankylosis	0.2% (n = 4)	0.0% (n = 0)	0.0% (n = 0)	0.2% (n = 4)

**Fig 2:**
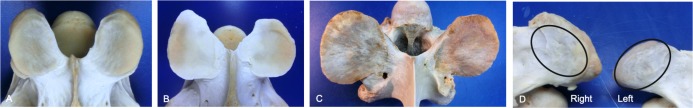
Progression in left-right asymmetry of the cranial articular processes (dorsal views). Normal morphology (A) is shown to illustrate changes associated with mild (B), moderate (C) and severe (D) grades of cranial articular process asymmetry. Note the >60% left-right difference in size (mirrored circles) within the severe grade (D, lateral view). Vertebrae, ages and breeds of horses: (A) C3 from a 6-year-old Warmblood, (B) C3 from a 5-year-old Thoroughbred, (C) T1 from a 7-year-old Warmblood, and (D) C7 from a 12-year-old Quarter Horse.

**Fig 3:**
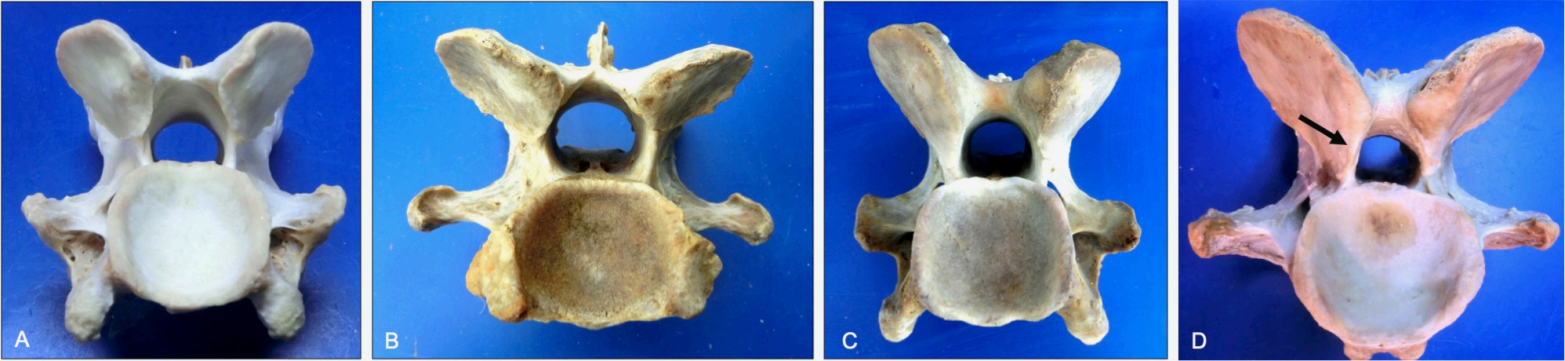
Progression in left-right asymmetry of the caudal articular processes (caudal views). Normal morphology (A) is shown to illustrate changes associated with mild (B), moderate (C) and severe (D) grades of caudal articular process asymmetry. Vertebral canal compromise was noted in some vertebrae with severely affected caudal articular processes (D, arrow). Vertebrae, ages and breeds of horses: (A) C6 from a 2-year-old Quarter Horse, (B) C7 from a 15-year-old Quarter Horse, (C) C6 from a 5-year-old Thoroughbred, and (D) C5 from a 7-year-old Warmblood.

Articular process asymmetry had an overall prevalence of 1% with left-right differences in articular surface area > 60% in severe cases (Fig 2). Surprisingly, even the most severe articular process enlargement did not impinge on the intervertebral foramen but did show evidence of vertebral canal compromise when the caudal articular process was affected (Fig 3).

**Fig 4:**
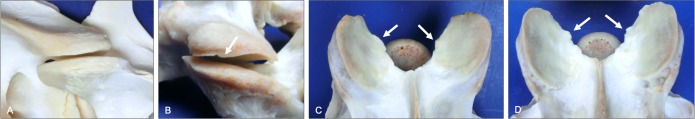
Progression in joint margin lipping (lateral and dorsal views). Normal morphology (A) is shown to illustrate changes associated with mild (B), moderate (C) and severe (D) grades of joint margin lipping. Note the progression of bony proliferation from a localized lesion (B) to more of a regional (C) and bilateral distribution pattern (D, arrows). Vertebrae, ages and breeds of horses: (A) C6-C7 from a 3-year-old Quarter Horse, (B) T1-T2 from a 28-year-old mixed breed, (C) C4 from a 2-year-old Quarter Horse, and (D) C5 from a 2-year-old Quarter Horse.

**Fig 5:**
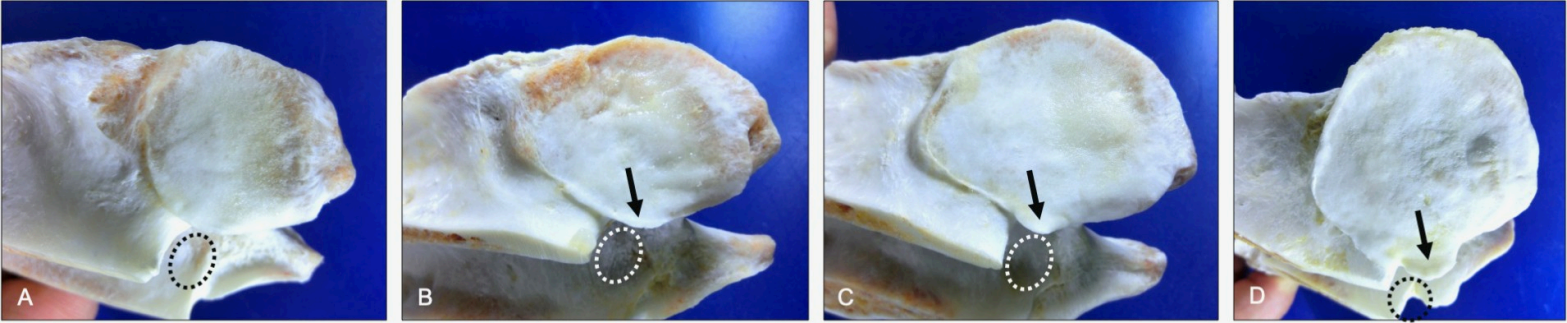
Progression in medial joint margin lipping (ventrolateral views). Normal morphology (A) is shown to illustrate changes associated with mild (B), moderate (C) and severe (D) grades of medial joint margin lipping of the caudal articular processes. Note the progression in medial expansion of the articular joint margins (arrows) into the region of the intervertebral foramen (dashed ovals). Vertebrae: (A) C3, (B) C4, (C) C5, and (D) C7 are all from a 12-year old Thoroughbred.

**Fig 6:**
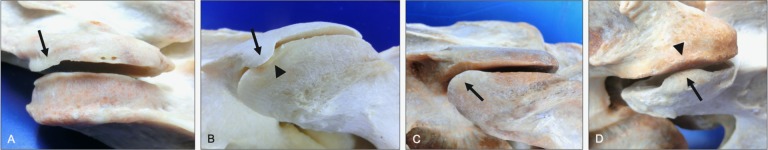
Progression of ventrally- or dorsally-deviated lateral joint margins (lateral views). Mild (A) and moderate (B) grades of ventrally-deviated lateral joint margins of the caudal articular processes. Mild (C) and moderate (D) grades of dorsally-deviated lateral joint margins of the cranial articular processes. Note the flattening of the articular joint margins (arrowheads) opposite to the overhanging bony proliferation (arrows). Vertebral levels, ages and breeds of horses: (A) C4-C5 from a 28-year-old mixed breed, (B) C3-C4 from a 5-year-old Thoroughbred, (C) C5-C6 from a 15-year-old Quarter Horse, and (D) C2-C3 from a 15-year-old Quarter Horse.

Joint margin lipping was found in 23% of all articular process and was mild in 21% of those cases (Table 1). Lipping occurred locally or in a regional or circumferential distribution pattern that formed irregular or saw tooth-shaped joint margins in moderate to severely affected articular processes (Fig 4). Lipping of the joint margins mostly affected the medial (30%) and lateral (29%) quadrants of the articular surfaces. As medial joint margin lipping progressed in size, the bony projection began to expand into the region of the intervertebral foramen (Fig 5). As the lipping increased in size laterally, the osseous projection became dorsally- or ventrally-deviated relative to the affected articular surface and formed a joint margin concavity (Fig 6).

**Fig 7:**
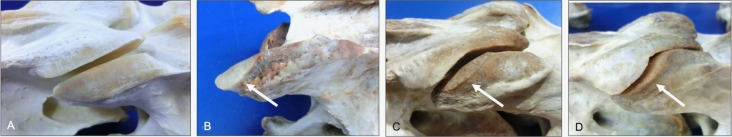
Progression in flattening of the lateral joint margins (lateral views). Normal morphology (A) is shown to illustrate changes associated with mild (B), moderate (C) and severe (D) grades of lateral joint margin flattening (i.e., regressive modeling) of the cranial articular processes (arrows). Note the concurrent gradations of overhanging lateral joint margins of the caudal articular processes (C and D). Vertebral levels, ages and breeds of horses: (A) C4-C5 from a 5-year-old Thoroughbred, (B) C6 from a 15-year-old Thoroughbred, (C) C3-C4 from a 13-year-old Appaloosa, and (D) C5-C6 from a 10-year-old Warmblood.

**Fig 8:**
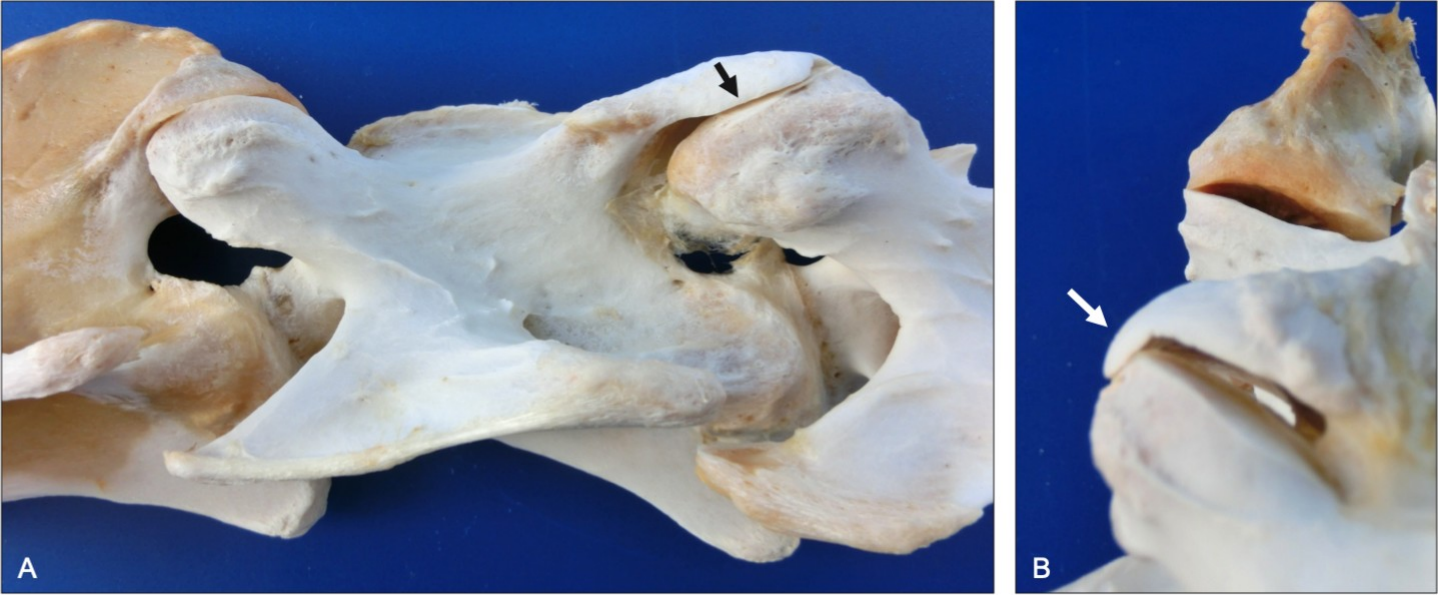
Lateral joint margin modeling (lateral and caudal views). Normal morphology at C2-C3 (A, left) is shown to illustrate changes associated with the prominent ventral projection of bone from the lateral joint margin of the left C3 caudal articular process (A right; arrow). Note the ventral projection of bone restricts mediolateral translation of the affected C3-C4 articular surfaces (B, bottom), compared to C2-C3 (B, top) from a 9-year-old Quarter Horse.

**Fig 9:**
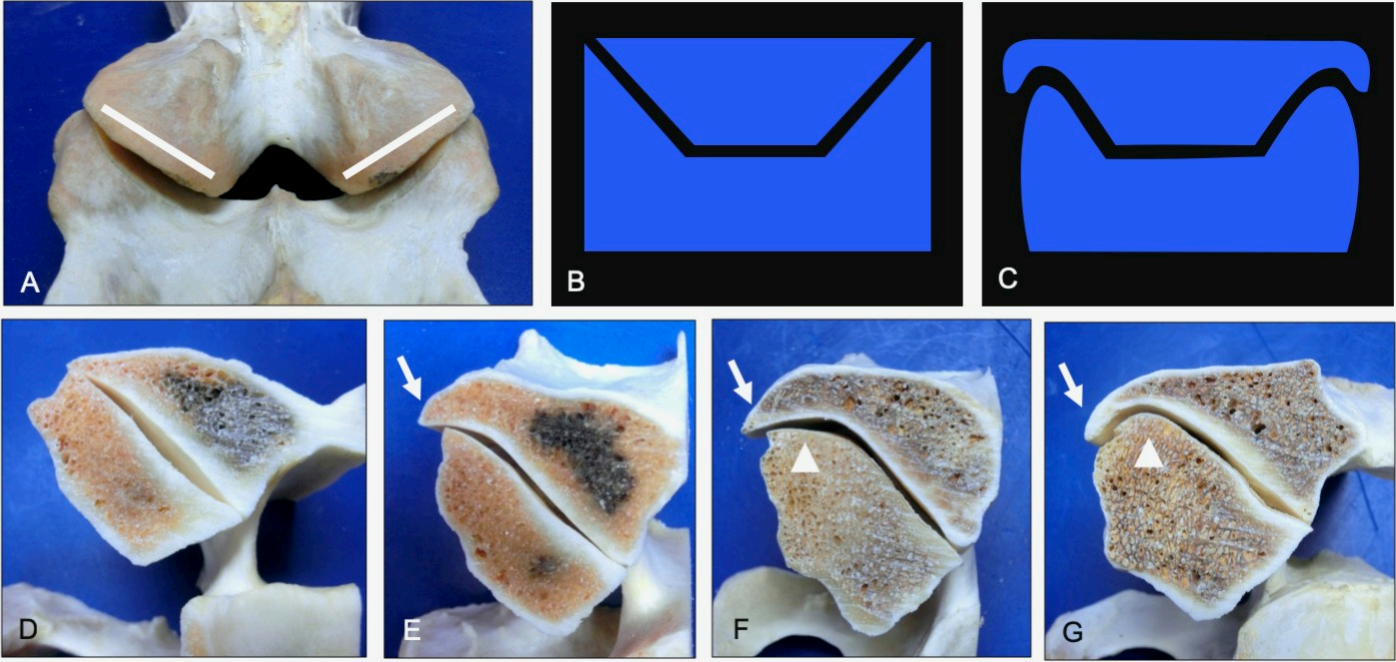
Progression in lateral joint margin modeling (transverse sections, caudal views). Caudal view of the normal articular process morphology of the caudal articular processes (A, lines) and models illustrating normal (B) and progressive modeling of the lateral joint margin (C). Normal morphology (D) is shown to illustrate changes from biplanar articular surfaces to prominent overhanging lateral joint margins associated with mild (E), moderate (F) and severe (G) grades of progressive modeling of the caudal articular processes (arrows) and concurrent regressive modeling of the paired cranial articular processes (arrowheads). Vertebral levels, ages and breeds of horses: (D) C4-C5 from a 12-year-old Quarter Horse, (E) C6-C7 from a 12-year-old Quarter Horse, (F) C5-C6 from a 9-year-old Quarter Horse, and (G) C3-C4 from a 9-year-old Quarter Horse.

**Fig 10:**
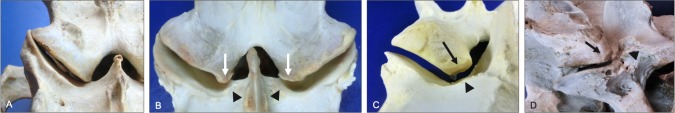
Progression in medial joint margin flattening associated with extension impingement (caudal views). Normal morphology (A) is shown to illustrate changes associated with mild (B), moderate (C) and severe (D) grades of medial joint margin flattening of the caudal articular processes (arrows) associated with impingement on the paired adjacent dorsal lamina (arrowheads). Note the severe osteophytic proliferation present on the opposing articular surface in the severe grade (D; caudolateral view). Vertebral levels, ages and breeds of horses: (A) C5-C6 from a 10-year-old Quarter Horse, (B) C6-C7 from a 2-year-old Quarter Horse, (C) C6-C7 from a 2-year-old Warmblood, and (D) C7-T1 from a 7-year-old Warmblood.

**Fig 11:**
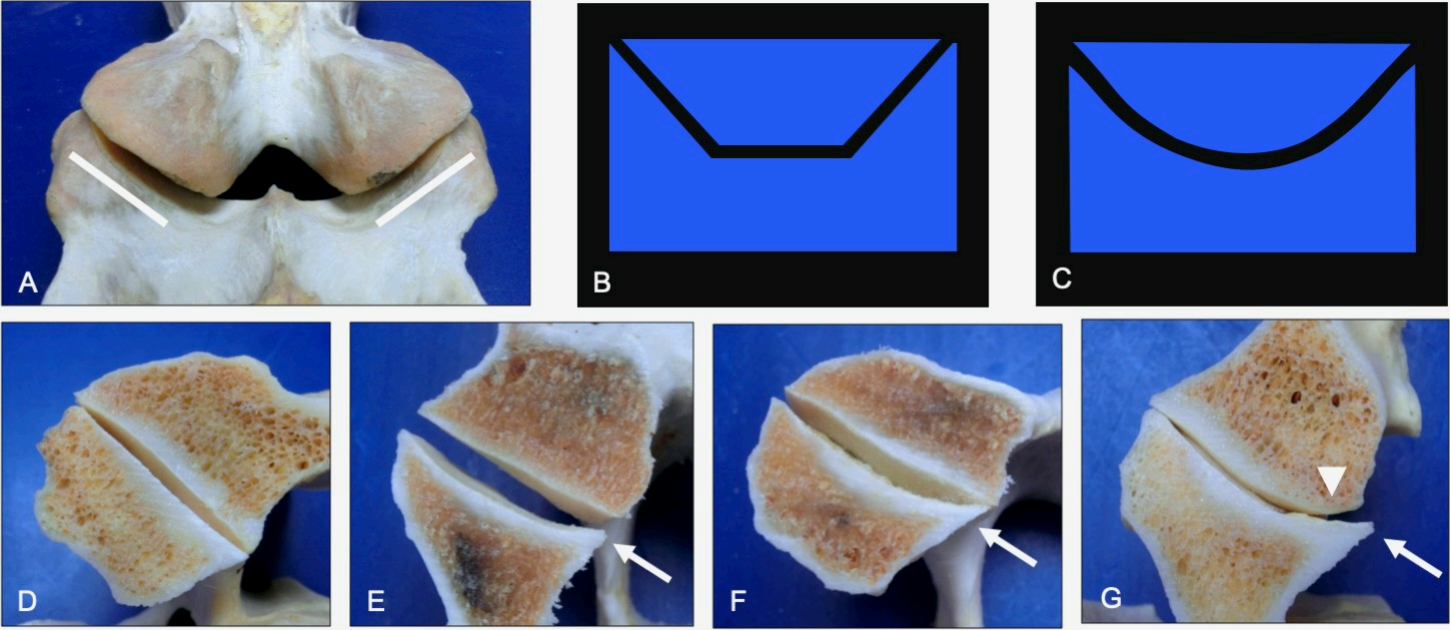
Progression in medial joint margin modeling (transverse sections, caudal views). Caudal view of the normal articular process morphology of the cranial articular processes (A, lines) and models illustrating normal (B) and progressive modeling of the medial joint margin (C). Normal morphology (D) is shown to illustrate changes from biplanar articular surfaces to articular surface concavity associated with mild (E), moderate (F) and severe (G) grades of progressive modeling of the cranial articular processes (arrows) and concurrent regressive modeling of the paired caudal articular processes (arrowhead). Vertebral levels, ages and breeds of horses: (D) C3-C4 from a 5-year-old Warmblood, (E) C2-C3 from a 15-year-old Quarter Horse, (F) C3-C4 from a 15-year-old Quarter Horse, and (G) C2-C3 from a 5-year-old Warmblood.

Flattening of the joint margins occurred in 24% of articular processes (Table 1). Lateral joint margin flattening of the cranial articular process typically occurred opposite to joint margin lipping or modeling to accommodate the abnormal, proliferative bony projecting from the paired articular surface (Fig 7). Modeling of the joint margins had an overall prevalence of 11% (Table 1). All affected articular processes with overhanging joint margins (i.e., progressive modeling) had concurrent flattening (i.e., regressive modeling) of the opposing lateral joint margin (Fig 8). All modeled articular joint margins had grossly evident hyaline cartilage that was contiguous with the planar surface articular cartilage. Modeling of the lateral joint margin occurred most commonly (38%) and appeared to be a more severe or progressive form of articular lipping that primarily affected the caudal articular processes where it formed an overhanging lateral projection of bone (Fig 9). In contrast to flattening of the lateral joint margins, medial joint margin flattening appeared to be produced by impact or impingement of the caudal articular process against the dorsal aspect of the paired articular surface during extreme end-range of intervertebral extension (i.e., extension impingement; Fig 10). Flattening (i.e., regressive modeling) of the medial joint margin had an overall prevalence of 4% and was localized to the cranial articular processes which produced a medial articular surface concavity (Fig 11).

**Fig 12:**
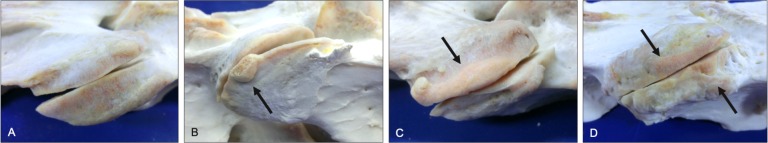
Progression in osteophyte development along the lateral joint margins (lateral views). Normal morphology (A) is shown to illustrate changes associated with mild (B), moderate (C) and severe (D) grades of lateral joint margin osteophytes (arrows). Note the generalized distribution of roughened, irregular joint margins and thickening of the paired articular processes in the severe grade (D). Vertebral levels, ages and breeds of horses: (A) C4-C5 from a 12-year-old Thoroughbred, (B) C6-C7 from a 2-year-old Quarter Horse, (C) C6-C7 from a 28-year-old mixed breed, and (D) C5-C6 from a 14-year-old Quarter Horse.

**Fig 13:**
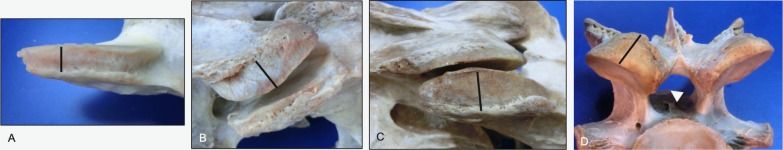
Progression in articular process thickening (lateral and caudal views). Normal morphology (A) is shown to illustrate changes associated with mild (B), moderate (C) and severe (D) grades of cranial and caudal articular process thickening (lines). Note the asymmetrical articular process thickness within paired cranial-caudal articular surfaces (B and C) and the left-right differences and mild vertebral canal compromise (arrowhead) in the severe grade (D). Vertebral levels, ages and breeds of horses: (A) C6 cranial articular process from a 28-year-old mixed breed, (B) C6-C7 from a 24-year-old Quarter Horse, (C) C4-C5 from a 13-year-old Appaloosa, and (D) C7 caudal articular process from a 7-year-old Warmblood.

**Fig 14:**
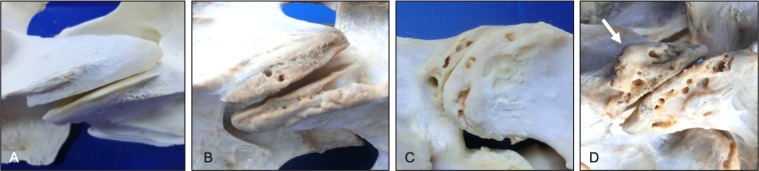
Progression in presence and severity of enlarged vascular channels (lateral views). Normal morphology (A) is shown to illustrate changes associated with mild (B), moderate (C) and severe (D) grades of enlarged vascular channels localized within the margins of the joint capsule. Note the large enthesophyte (arrow) associated with the joint capsule insertion site in the severe grade (D). Vertebral levels, ages and breeds of horses: (A) C5-C6 from a 3-year-old Quarter Horse, (B) C5-C6 from a 19-year-old Morgan, (C) C6-C7 from a 5-year-old Quarter Horse, and (D) C7-T1 from a 19-year-old Morgan.

**Fig 15:**
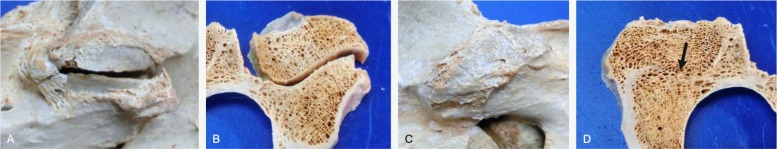
Partial and complete ankylosis of the articular processes. Lateral views of partial (A) and complete (C) ankylosis and corresponding transverse sections (B and D) of the C2-C3 articular processes. Note that with partial ankylosis the ossification is mostly limited to the joint capsule margins (A). On the transverse section of complete ankylosis (D), there is resorption of the subchondral bone plates and replacement with trabecular bone (arrow).

Osteophytes were the most common type of bony lesion (24%) affecting the articular processes and had the highest prevalence of moderate (9%) to severe (2%) grades of severity (Table 1). Osteophytes initially occurred as small nodules along the periarticular joint margins and progressed to more expansive bony proliferation and extensive articular process modeling (Fig 12). Grossly thickened articular processes with smooth periarticular bone (i.e., no evidence of osteophytes) had an overall prevalence of 3% (n = 59) and was noted in both paired and unpaired articular processes (Fig 13). Enlarged vascular channels was noted in 3% (n = 47) of articular processes and often occurred in conjunction with moderate to severe osteophytes (Fig 14). The overall prevalence of combined osteophytes and enthesophytes present within a single affected articular process was 13% (n = 60) out of 482 articular processes that had either osteophytes (n = 442) or evidence of enthesopathies (n = 160). Bilateral ankylosis of the C2-C3 articular processes was identified in a single horse. On transverse section, variable degrees of subchondral bone plate resorption were noted in conjunction with the ankylosis (Fig 15).

**Fig 16:**
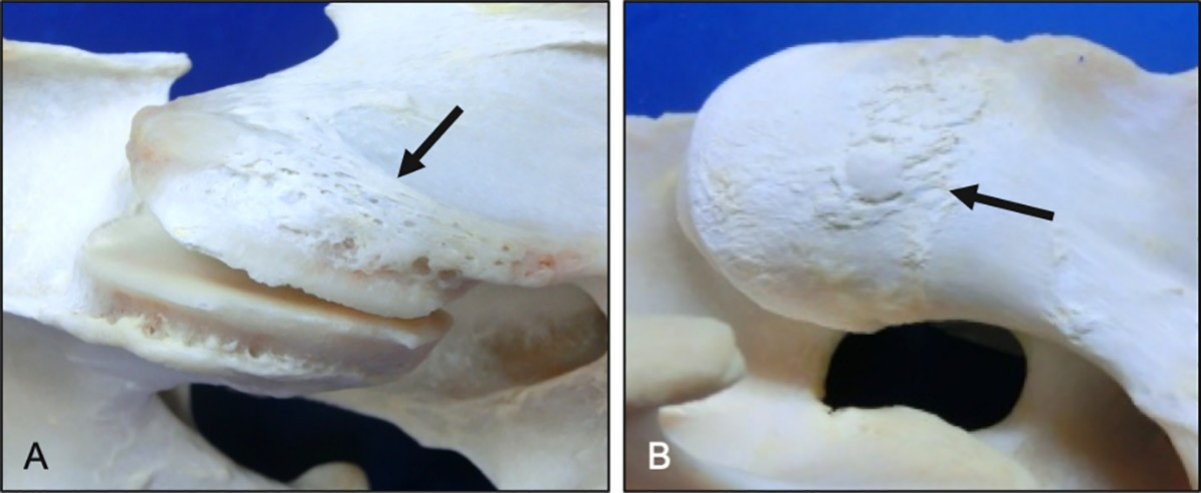
Progression in periosteal callus (lateral views). Raised areas of woven bone (arrows) located at joint capsule (A) and intertransverse muscle attachment sites (B). Vertebral levels, ages and breeds of horses: (A) C4-C5 from a 17-year-old Paint, (B) C3-C4 from a 5-year-old Warmblood.

**Fig 17:**
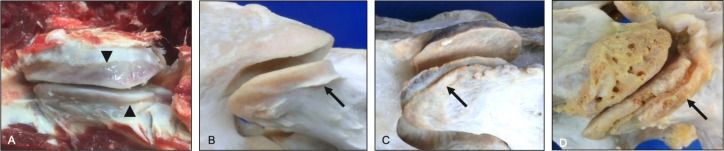
Progression in joint capsule enthesophytes (lateral views). Normal joint capsule insertion sites (A) have no evidence of ossification (arrowheads). Note the osseous progression from a mild, local sharp line (B), to a moderate regional, blunt enthesophyte (C), and finally to severe circumferential (D) joint capsule enthesophytes (arrows). Vertebral levels, ages and breeds of horses: (A) C4-C5 from a 5-year-old Quarter Horse, (B) C3-C4 from a 6-year-old Warmblood, (C) C5-C6 from a 19-year-old Morgan, and (D) C7-T1 from a 31-year-old Paint.

**Fig 18:**
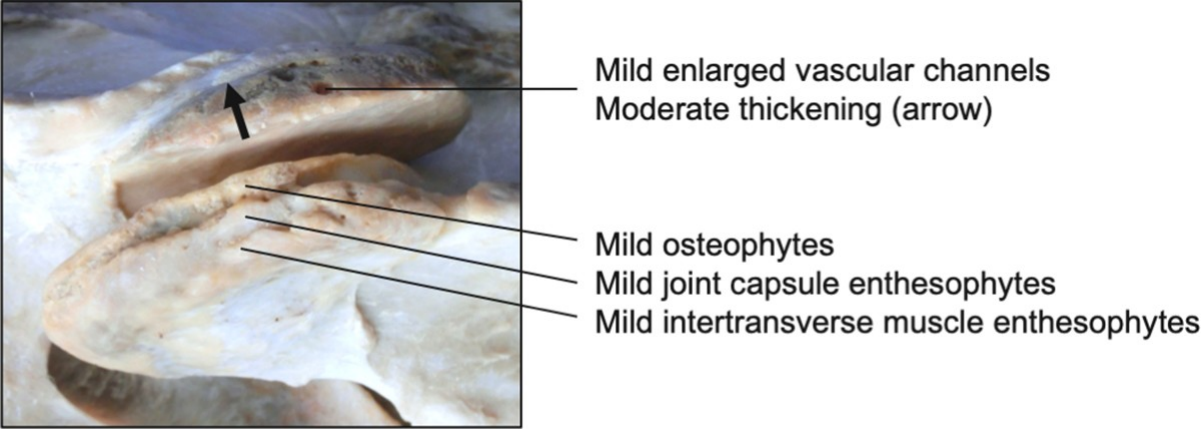
Combined osseous changes affecting the articular processes (dorsolateral view). Paired articular processes from a 19-year-old Morgan that shows evidence of moderate thickening (arrow) and mild enlarged vascular channels on the caudal articular process and a combination of mild grades of osteophytes along the lateral joint margin and enthesophytes of the joint capsule and intertransversarius muscle insertion sites on the cranial articular process.

Periosteal callus (i.e., woven bone) was seen in 1% (n = 11) articular processes and was located near joint capsule or muscle insertion sites (Fig 16). Joint capsule entheses occurred in 6% (n = 105) of articular processes and was mild in 3% (n = 47) and moderate in 3% (n = 51) specimens (Table 1). Joint capsule entheses appear to begin as small linear bony projections and progress to extensive periarticular thickening of the joint capsule insertion sites (Fig 17). A combination of osseous changes was often present in moderate to severely affected articular processes (Fig 18). The prevalence and types of combined osseous changes varied across vertebral levels, but within affected articular processes a single osseous change was noted in 64%, two combined osseous changes occurred at 32% of sites, and 4% of articular processes had 3 or 4 combined osseous changes present.

**Fig 19:**
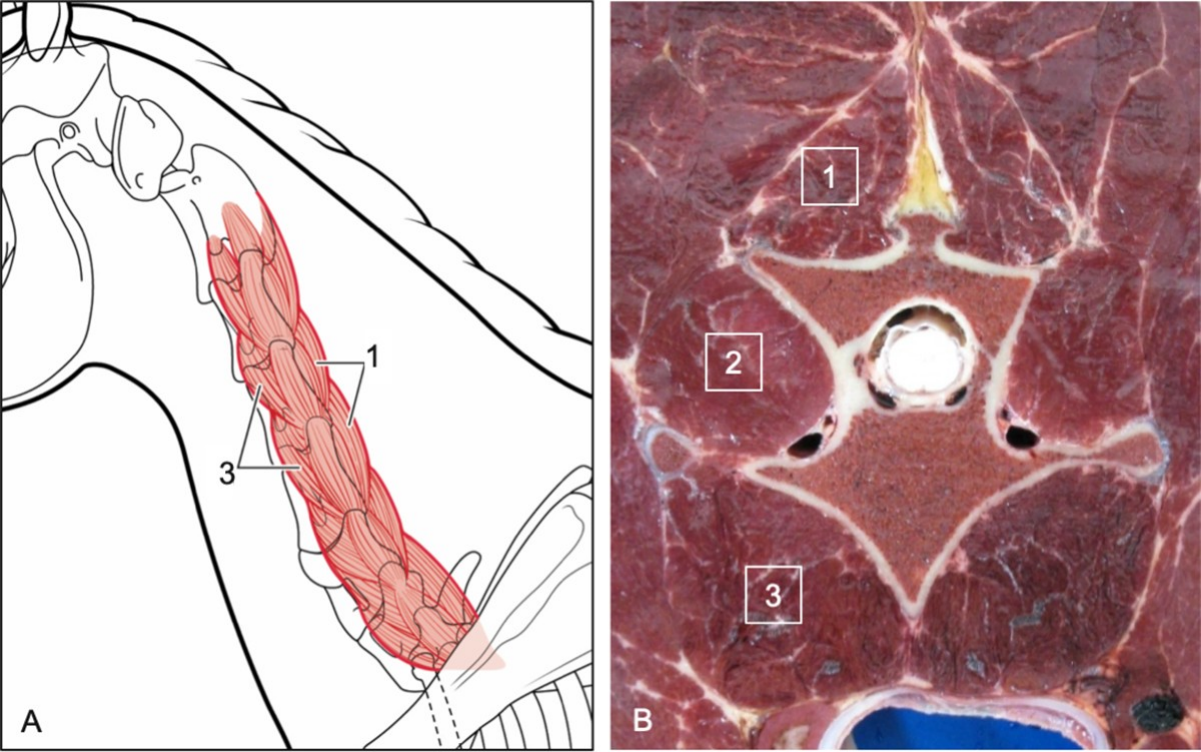
Deep cervical musculature. Illustration of the deep cervical musculature (A) and a transverse section of the cervical region (B) depicting the dorsally-located multifidi muscles (1), intertransversarius muscles laterally (2), and the longus colli (3) musculature along the ventral aspect of the vertebral bodies.

**Fig 20:**
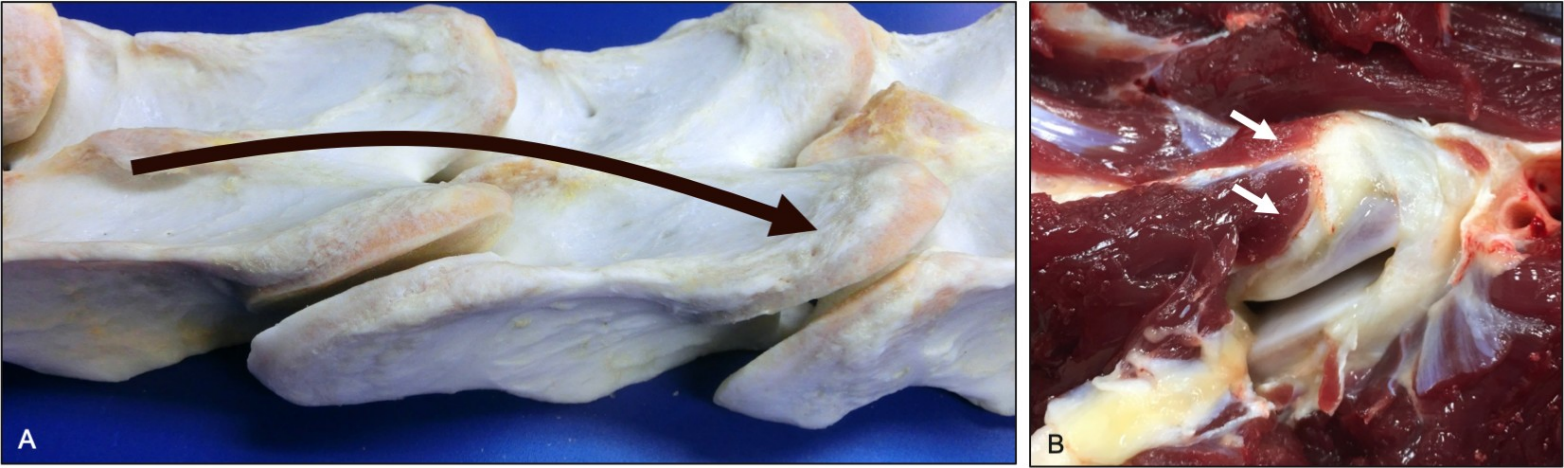
Multifidus muscle attachment sites (dorsolateral views). Illustration (A) of the general path of multifidus muscle orientation and representative attachment sites at the spinous processes and dorsal surface of adjacent caudal articular processes (curved arrow). Photograph (B) of the insertion of a multifidus muscle onto the dorsal surface of a caudal articular process (arrows).

**Fig 21:**
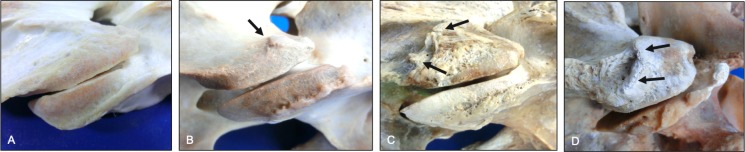
Progression in multifidus muscle enthesophytes (dorsolateral views). Normal morphology (A) is shown to illustrate changes associated with mild (B), moderate (C) and severe (D) grades of multifidus muscle enthesophytes (arrows) on the dorsal aspect of caudal articular processes. Vertebral levels, ages and breeds of horses: (A) C3-C4 from a 12-year-old Thoroughbred, (B) C6-C7 from a 5-year-old pony, (C) C6-C7 from a 22-year-old Percheron, and (D) C7-T1 from a 22-year-old Percheron.

**Fig 22:**
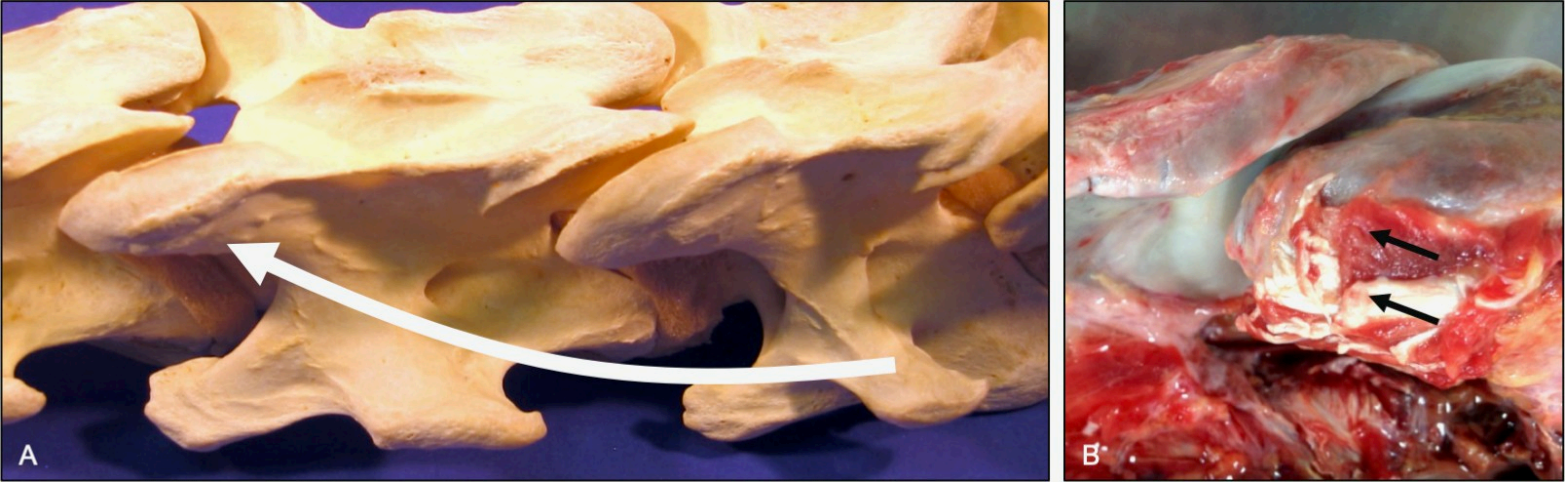
Intertransverse muscle attachment sites (dorsolateral and ventrolateral views). Illustration (A) of the general path of intertransverse muscle orientation and representative attachment sites at the transverse processes and ventral surface of adjacent cranial articular processes (curved arrow). Photograph (B) of the insertion of an intertransverse muscle onto the ventral surface of a cranial articular process (arrows).

**Fig 23:**
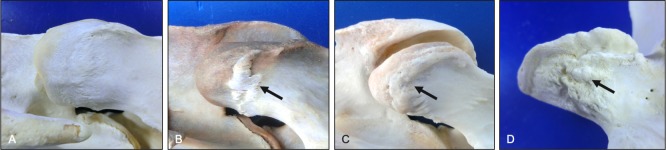
Progression in intertransverse muscle enthesophytes (ventrolateral views). Normal morphology (A) is shown to illustrate changes associated with mild (B), moderate (C) and severe (D) grades of intertransverse muscle enthesophytes (arrows) on the ventral surface of the cranial articular processes. Vertebral levels, ages and breeds of horses: (A) C5-C6 from a 3-year-old Quarter Horse, (B) C4-C5 from a 5-year-old pony, (C) C5-C6 from a 9-year-old Quarter Horse, and (D) T1 from a 2-year-old Warmblood.

The deep cervical musculature consists of a series of segmental muscles that attach dorsally (multifidi), laterally (intertransversarius), and ventrally (longus colli) (Fig 19). The cervical multifidi muscles have attachment sites at the dorsal spinous processes and caudal articular processes and span single to multiple vertebral levels (Fig 20) [36]. Multifidi muscle entheses were localized to the dorsal aspect of the caudal articular processes (Fig 21). The intertransverse muscles are located laterally in the cervical region and connect transverse processes and the ventral aspect of the cranial articular processes (Fig 22). Intertransverse muscle entheses were identified along the ventral surface of cranial articular processes (Fig 23).

### Prevalence and severity of bony changes

**Fig 24:**
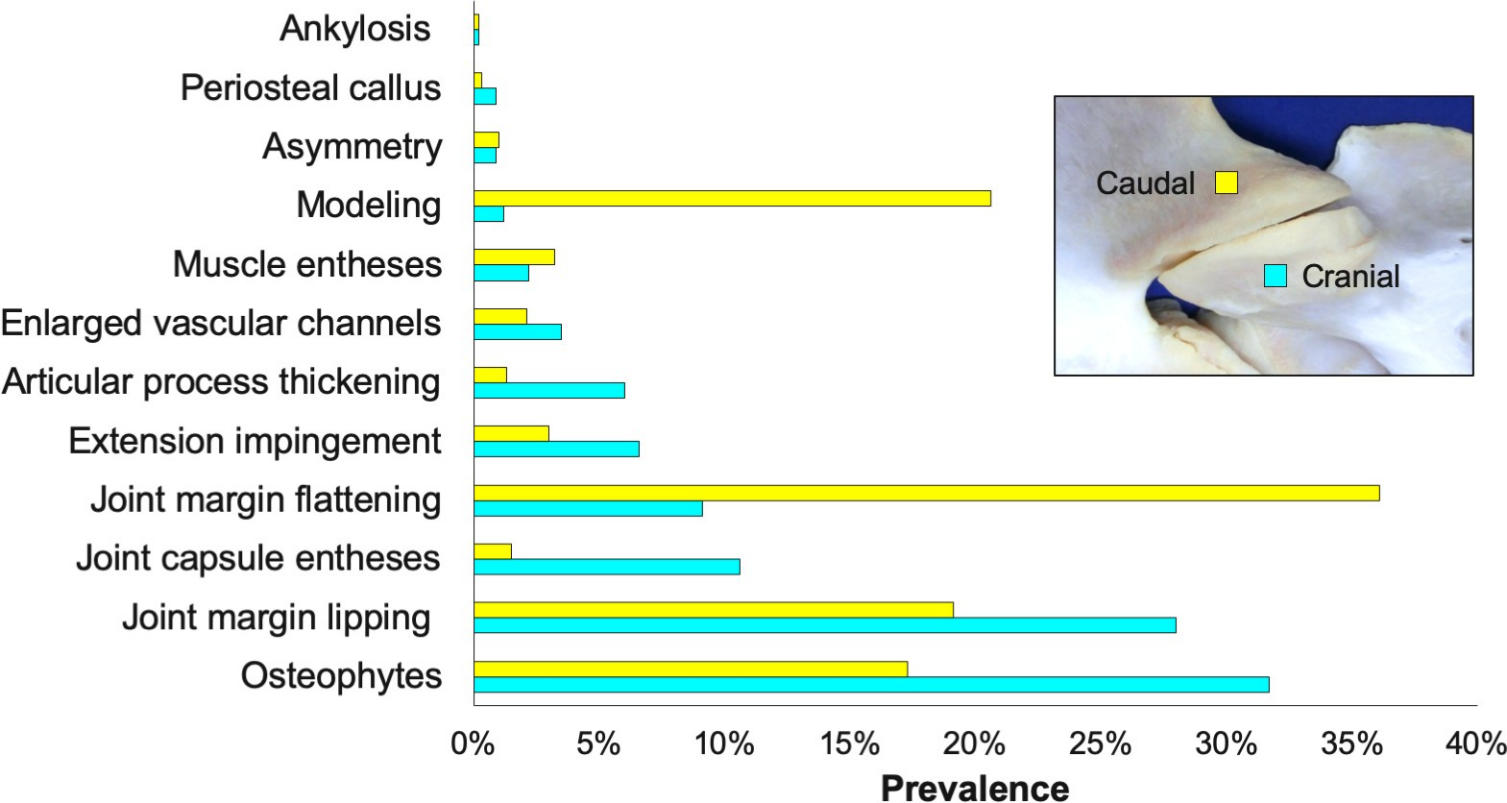
Prevalence of osseous changes. Prevalence of the types of bony changes that are localized to the cranial (blue) and caudal (yellow) articular processes across all subjects.

The overall prevalence of bony changes found within the cranial versus caudal articular processes was substantially different for several types of lesions (Fig 24). Within the cranial articular processes, the leading types of bony changes included osteophytes, lipping and joint capsule enthesis. The most prevalent types of bony changes found in the caudal articular processes were flattening and modeling.

**Fig 25:**
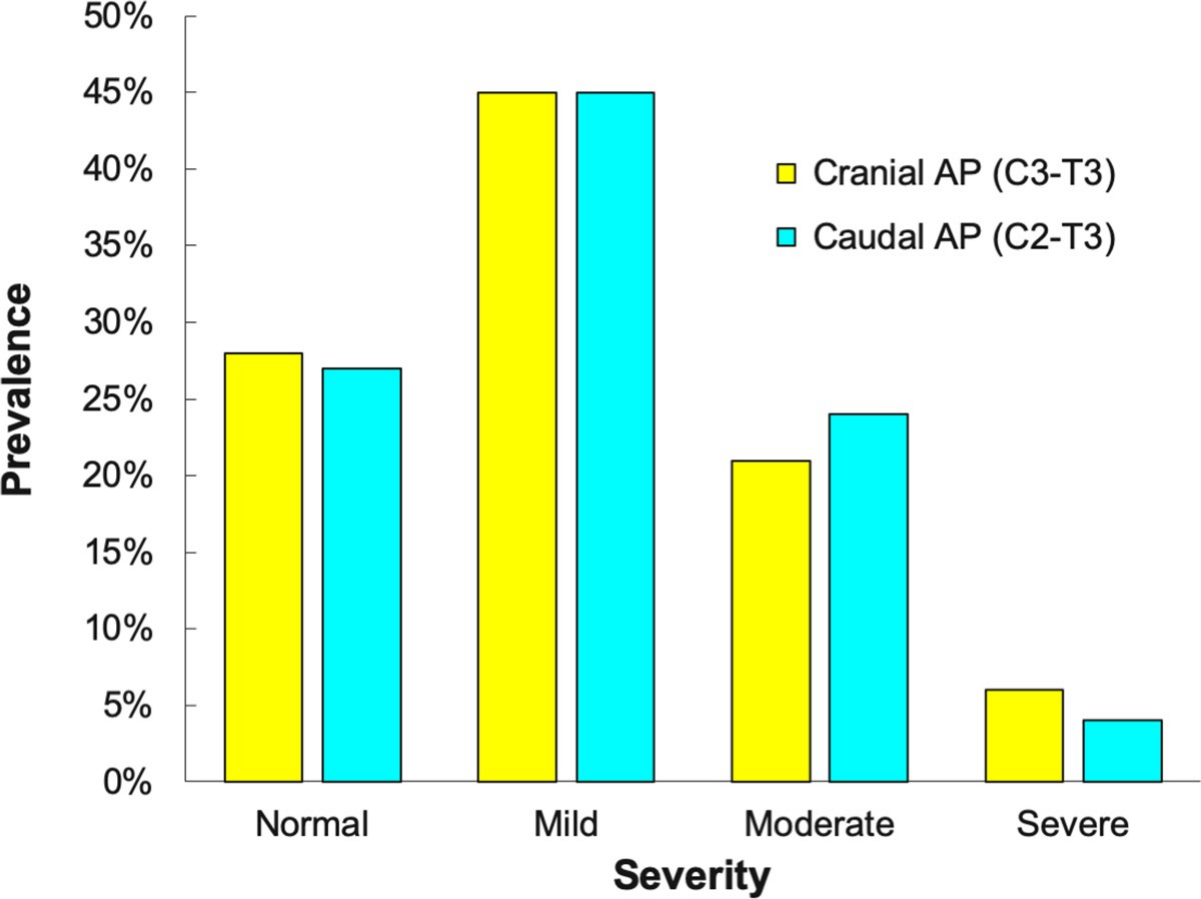
Frequency distribution of the severity of osseous changes. Prevalence of normal, mild, moderate and severe grades of osseous changes within the cranial (yellow) and caudal (blue) articular processes (AP) across all subjects.

Articular processes were graded as normal in 28% (n = 516) of locations, had mild changes in 45% (n = 836), moderate changes in 22% (n = 415), and severe changes in 5% (n = 86) of articular processes. The grades of severity across the different types of lesions did vary between the cranial versus caudal articular processes for some of the osseous changes (S1 Table). Osteophytes, lipping, joint capsule entheses and articular process thickening all were more severe within the cranial articular processes; whereas, the caudal articular process had more severe flattening and modeling of the joint margins. Although, the overall grades for all osseous changes were similar within the cranial versus caudal articular processes (Fig 25).

### Quadrant locations of bony changes

Within the different quadrants of the articular joint margins, the prevalence of osseous changes was highest for the medial and lateral joint margins (Table 2). Osteophytes were observed more commonly in the cranial quadrant and periarticular locations.

**Table 2:** Prevalence of bony changes within each articular quadrant.

Osseous changes	Cranial	Caudal	Medial	Lateral	Periarticular
Osteophytes	63%	29%	15%	3%	49%
Lipping	22%	8%	30%	29%	11%
Flattening	10%	18%	50%	8%	0%
Modeling	0%	1%	0%	38%	1%
Joint capsule enthesis	0%	14%	0%	12%	2%
Thickening	0%	0%	0%	0%	23%
Extension impingement	0%	24%	3%	0%	0%
Enlarged vascular channels	3%	5%	2%	0%	6%
Intertransverse muscle enthesis	0%	0%	0%	8%	0%
Asymmetry	0%	0%	0%	0%	7%
Periosteal callus	0%	1%	1%	0%	0%
Ankylosis	0%	0%	0%	0%	2%
**Pooled**	11%	14%	35%	27%	13%

When the cranial and caudal articular processes are assessed separately, the cranial articular processes had a higher prevalence of bony changes affecting the cranial-caudal joint margins; whereas, in the caudal articular processes the medial-lateral joint margins were more affected (S2 and S3 Tables).

### Prevalence of bony changes by vertebral level

The prevalence of the different types of bony changes varied across vertebral levels (Table 3). Ankylosis was only noted at C2-C3. From C3-C6, lipping, modeling, thickening and joint capsule enthesis occurred more commonly. At C6-T2, osteophytes, flattening, extension impingement, and enlarged vascular channels were the more common types of bony changes. Articular process asymmetry was most prevalent at T2-T3.

**Table 3:** Prevalence of bony changes within an articulation at each vertebral level.

Osseous changes	C2-C3	C3-C4	C4-C5	C5-C6	C6-C7	C7-T1	T1-T2	T2-T3	T3-T4
Osteophyte	30%	12%	17%	16%	30%	45%	32%	17%	11%
Flattening	25%	20%	21%	25%	35%	37%	34%	1%	1%
Lipping	20%	38%	47%	30%	18%	11%	15%	18%	7%
Modeling	16%	21%	16%	8%	14%	7%	8%	2%	-
Joint capsule enthesis	5%	11%	12%	11%	5%	2%	1%	-	-
Thickening	-	7%	7%	2%	5%	4%	-	-	-
Extension impingement	1%	-	-	2%	5%	27%	4%	-	1%
Enlarged vascular channels	-	-	1%	5%	5%	8%	2%	-	-
Muscle enthesis	2%	3%	4%	2%	5%	5%	-	-	-
Asymmetry	-	-	-	-	-	-	1%	4%	4%
Periosteal callus	-	-	-	2%	-	1%	-	-	3%
Ankylosis	2%	-	-	-	-	-	-	-	-

0% prevalence (-)

When the cranial articular processes are assessed, osteophytes were more common at C3 and T1-T2 and extension impingement and enlarged vascular channels occurred more frequently at T1 (S4 Table). Within the caudal articular processes, flattening occurred more commonly at C2 and C5-T1 and modeling occurred more commonly at C2-C6 (S5 Table).

### Severity of bony changes by vertebral level

The severity of bony changes varied across vertebral levels (Table 4). The highest prevalence of mild changes was localized to the C3-T2 vertebral levels; moderate changes at C6-T2; and severe changes were noted more commonly at C2-C3 and C6-T2.

**Table 4:** Vertebral level distribution of the prevalence of grades of severity within an articulation.

Vertebrae	Normal	Mild	Moderate	Severe
C2-C3	25%	50%	19%	7%
C3-C4	16%	57%	24%	3%
C4-C5	15%	60%	22%	3%
C5-C6	27%	53%	17%	4%
C6-C7	20%	48%	27%	5%
C7-T1	9%	38%	41%	12%
T1-T2	25%	46%	25%	5%
T2-T3	61%	25%	12%	1%
T3-T4	81%	12%	5%	3%

When cranial and caudal articular processes are assessed independently, moderate changes occurred more commonly in the caudal articular processes at the C6-T1 vertebral levels (Table 5). Severe changes in the cranial articular processes were found more at the C7-T1 vertebral levels. Pooled values for the grades of severity were quite similar between cranial and caudal articular processes within a synovial articulation.

**Table 5:** Vertebral level distribution of the prevalence of grades of severity at the cranial and caudal Articular Processes (AP).

Caudal AP					Cranial AP				
Vertebra	Normal	Mild	Moderate	Severe	Vertebra	Normal	Mild	Moderate	Severe
**C2**	29%	51%	17%	5%	**C3**	23%	48%	21%	8%
**C3**	20%	51%	25%	4%	**C4**	13%	63%	23%	2%
**C4**	13%	65%	22%	1%	**C5**	18%	55%	23%	5%
**C5**	25%	57%	15%	3%	**C6**	28%	49%	19%	4%
**C6**	11%	50%	34%	5%	**C7**	30%	46%	20%	4%
**C7**	3%	45%	45%	8%	**T1**	16%	31%	38%	16%
**T1**	13%	49%	35%	3%	**T2**	37%	42%	14%	7%
**T2**	58%	25%	15%	2%	**T3**	64%	28%	9%	0%
**T3**	81%	12%	5%	3%					

### Paired cranial-caudal articular process grades

When comparing the grades of severity of bony changes between the cranial and caudal articular processes within individual synovial articulations, 81% (13 of 16) of paired articular surfaces did not have significantly different grades of severity (S6 Table). The T1-T2 synovial articulations had more severely affected caudal articular processes, compared to the paired cranial articular processes. Pooled across all vertebral levels, cranial-caudal severity scores were the same for 52% of synovial articulations.

### Left-right articular process grades

Left-right comparisons of the severity of articular process changes were not significantly different in 82% (14 of 17) of articular processes (S7 Table). Pooled across all vertebral levels, the severity of paired left-right articular process changes was equal in 68% of paired articulations. Therefore, osseous changes are more severe unilaterally in approximately 30% of cases; however, there was not a consistent left-right prevalence across vertebral levels.

### Unilateral cranial-caudal articular process grades

Comparing the severity of unilateral bony changes between the cranial and caudal articular processes within a single vertebra, 56% (9 of 16) comparisons were significant or had a trend to be different across the C3 to T3 vertebral levels (S8 Table). The clustering of significant results at the C6-T1 vertebral levels likely reflects the higher overall prevalence of moderate to severe changes identified within this region (Tables 4 and 5). Pooled across all vertebral levels, the grade of severity of unilateral (i.e., left or right sides) bony changes of the cranial and caudal articular processes within a vertebra was the same in 55% of vertebrae, which did not clearly support or refute the premise of a “domino effect” within the cervical vertebral region.

### Signalment relationships with severity of bony changes

Age and wither height were positively associated with grades of increased severity of bony changes within 61% (11 of 18; rho = 0.29 to 0.58) and 78% (14 of 18; rho = 0.28 to 0.47) of paired synovial articulations, respectively. Compared with females, male horses had significantly increased severity of bony changes at 11% (2 of 18; rho = 0.28) of paired synovial articulations.

## Discussion

An overall prevalence of 72% bony changes localized to the articular processes was found in this mixed population of horses with unknown histories of neck pain or dysfunction, which is higher than a previous study that found 48% of clinically normal (i.e., non-ataxic) horses had moderate to severe degenerative changes of the articular processes [21]. In our subjects, the highest prevalence of abnormal bony changes localized to a vertebral level was 91% recorded at C7-T1. Additionally, a prevalence of ≥ 75% was found at C2-C3, C6-C7 and T1-T2. Several important factors need to be considered when referring to these prevalence rates: 1) this was a gross anatomic study, which allows better visualization of bony changes compared to diagnostic imaging; 2) the prevalence includes a wide variety of bony changes other than commonly reported osteophytes; 3) there was a high prevalence of mild grades of disease in this study; 4) the clinical significance of the changes is not known; 5) it is possible that some of the horses had signs of neck pain or stiffness as they did not have a thorough physical examination of the cervicothoracic region prior to death; 6) there are certainly some types of boney changes that are more clinically-relevant or more readily visualized on diagnostic imaging than other types; and 7) few prior studies have reported findings within the entire cervical region due to a perceived lack of clinical relevance of bony changes within the upper cervical regions or have not included the cranial thoracic region due to difficulties in clinical examination and diagnostic imaging within this spinal region.

### Signalment

Older and taller horses had a significantly higher prevalence of severe grades of bony changes, which is consistent with previous studies [24] and supports the experimental hypothesis. A positive age association would be expected if bony changes were developmental and cumulative over the life of the individual horse [37]. Interestingly, it has been shown that old horses require more muscle activity to support the neck against the effects of gravity [38], which may increase joint compression. The association between increased wither height (i.e., larger body mass) contrasts with the situation in humans in which height and body mass are not correlated to the presence or severity of cervical pathology [39]. The difference between horses and humans is likely a consequence of the more horizontal orientation and greater length of the equine neck, which acts as a cantilevered beam [40]. The mass of the head and cranial vertebral segments of the neck exert increased forces on the more caudal vertebrae that form the cervicothoracic junction. Larger horses have heavier cranial neck segments and a longer lever arm formed by the increased length of their neck, both of which likely contribute to the higher prevalence of osseous changes found in taller horses [38].

A higher prevalence of articular process enlargement has been reported in male horses, which was likely associated with their larger body mass compared to mares [19]. However, in our study male horses had significantly higher grades of severity of bony changes at only two synovial articulations (i.e., C2-C3 and C6-C7). We did not evaluate the prevalence or grades of severity between horse breeds as we used a diverse population of numerous breeds with low numbers in most breed categories. Performance records were incomplete, so it was not possible to evaluate the effect of athletic discipline on bony changes.

### Severity of bony changes

The grading system used in this study to score the severity of bony changes localized to the articular processes was a composite of methods used to assess gross cervical pathology in humans [37, 41] and radiographic abnormalities in horses [12]. Simple grading systems referencing normal (or absent), mild, moderate, or severe changes seem to be the most useful approach to categorize osteophytes or enthesophytes within the cervical vertebral region and met our needs in capturing the various types and progression of bony changes. Elaborate scoring systems that attempt to compile various combinations of bony changes into different categories or to grade the patency of intervertebral foramen on lateral radiographs are likely inaccurate or misleading [24].

### Quadrant locations of bony changes

The medial and lateral joint margins were most commonly affected in the caudal articular processes, which has relevance in the interpretation of diagnostic images of the cervical region. The lateral joint margins are most readily visualized with ultrasonography and bony proliferation in this region would be most evident dorsal to the articular processes on lateral radiographs. The medial joint margin changes are likely difficult to identify due to summation effects but might be better captured on oblique radiographic views. The medial-lateral distribution of bony changes may reflect increased biomechanical forces acting on these joint margins during coupled lateral bending and axial rotation of the cervical vertebrae [42]. In severe grades of articular process modeling, the large lateral overhanging joint margins appear to dramatically limit intervertebral motion, especially lateral bending and axial rotation.

Flexion and extension of the cervical vertebrae produces large compressive forces on the articular processes. Due to incongruent articular surfaces, there is increased loading within the cranial portion of paired articular processes during neck flexion and during extension there is increased loading within the caudal portion of the articular surfaces [40]. This loading pattern may account for the higher prevalence of osteophytes in the cranial quadrant of the articular surfaces in horses that spend more time with their neck in flexion (i.e., grazing). In contrast, ridden horses with their lower cervical region held in extension preferential load the caudal aspect of the articular processes, which may predispose to the development of caudal osteophytes and medial flattening due to articular process impingement.

Intervertebral foramen dimensions are reported to be reduced in horses with cervical myelopathy [1]. We did not find any evidence of substantial intervertebral foramen comprise or occlusion in our subjects; however, we could hypothesize that bony proliferation at the ventral or medial joint margins could have been interpreted as partial occlusion of the intervertebral foramen in some specimens if viewed on lateral radiographic images or during cervical extension [19, 43]. In specimens that had severe ventral or medial joint margin osteophytes, a clearly defined intervertebral osseous channel was still present at the caudal aspect of the intervertebral foramen for transmitting the spinal nerve and associated vasculature, which would likely be obscured on routine lateral radiographic images [9]. We did find evidence of bony proliferation that changed the shape of the dorsolateral vertebral canal, which had the potential to contribute to the development of cervical myelopathy [1]. However, articular process enlargement, when present, cannot necessarily be assumed to be the primary cause of any noted neurological deficits [24].

### Prevalence and severity of bony changes by vertebral level

The directions (e.g., flexion-extension) and ranges of joint motion that normally occur within the cervicothoracic intervertebral articulations are likely to affect the prevalence, location within an articular process, and the severity of osseous lesions [40]. Osteoarthritic changes within the cervical region have been most commonly reported at the C5-C7 vertebral levels [30, 31]. In our study, we found a large variety of different types of bony changes distributed from C2 to T3. Osteophytes were more prevalent at C6-T2, but also had a similar prevalence of 30% at C2-C3, which corresponds with the cranial and caudal extremities of the neck that undergo large ranges of joint motion and which may incur an increased risk for microtrauma in horses with constrained (e.g., draw reins) or chronically-induced and repetitive excessive head and neck postures.

The cervical spine is considerably stiffer in extension compared to other directions of joint motion [44]. The increased stiffness in extension is likely due to overlap and impingement of the cervical articular processes, which was noted in specimens with medial joint margin flattening of the caudal articular processes at the most commonly affected C6 to T1 vertebrae. The C7-T1 articulation had the highest prevalence and severity of bony changes in our study. This is in contrast to other reports that suggest that the C5-C7 vertebral levels are the most clinically relevant [25]. Computer modeling of the equine neck has confirmed that joint moments are highest at the base of the neck and C5–C6 is considered a functionally ‘low motion, high pressure’ joint [40]. As such, cervical vertebrae exposed to the larger joint moments over smaller joint surfaces are predisposed to a higher prevalence of osteoarthritis. However, due to the wide variety of bony changes distributed throughout the entire cervicothoracic region in our study, investigators need to consider including the entire cervical and cranial thoracic region as sources of neck pain or dysfunction in future projects.

### Articular process comparisons

There were significant differences in the prevalence of osseous changes localized to the cranial versus caudal articular processes, which implies that they have distinct and different anatomical features or biomechanical forces acting on the cranial and caudal articular processes [42]. These topographical differences are likely due to segmental motion patterns that induce localized and specific osteochondral forces or aberrant chemical signaling that initiates endochondral ossification at the joint margins or local entheses [45]. Bone can change its shape, density and orientation in response to biomechanical demands, which in turn can affect cervical vertebral motion and stability by limiting the direction and amount of segmental movement [40, 46]. It is likely that some horses with severe proliferative bony lesions had severely restricted intervertebral motion and overall neck stiffness.

The most common bony changes found at the cranial articular processes included osteophytes, lipping and joint capsule enthesophytes. It was surprising that osteophytes and joint capsule enthesophytes were not more evenly distributed between opposing articular surfaces as biomechanical or chemical mediators would be expected to similarly influence paired articular structures within a synovial articulation. However, the grades of severity were not significantly different in the majority of paired articular surfaces within an articulation. The most prevalent bony changes affecting the caudal articular processes included flattening and modeling to accommodate bony proliferation on the opposing articular surface. Flattening was mostly due to medial impingement on the adjacent articular process, which was most evident during extension of the affected vertebrae.

Left-right comparisons of the severity of bony changes were not significantly different at most vertebral levels, which supports the premise that biomechanical factors do act bilaterally in the cervical vertebral region [42]. Unilateral lesions would suggest the presence of a local inflammatory process or developmental defect (e.g., osteochondrosis). Intersegmental instability or stiffness at one intervertebral articulation is likely to induce compensatory movement patterns or osteoarthritis at adjacent vertebral levels; however, we did not find evidence of a domino-effect at the C5-T3 vertebral levels based on the evaluation of the severity of bony changes.

### Pathogenesis of bony changes

Osteophyte formation is initiated by the proliferation of progenitor cells at joint margins, followed by differentiation of these cells into chondrocytes that hypertrophy and form cartilage spurs that eventually undergo endochondral ossification [46]. Repeated microtrauma appears to interrupt this process so that the traumatized lipping at the joint margin becomes a series of irregular nodules of cartilage and bone [45]. Bony proliferation typically begins at a single site and may expand circumferentially along a joint margin to produce severe proliferation and deformation of the entire articular process. This sequence of events could readily explain the development of boney expansion within specific quadrants of the articular surfaces and would provide a mechanism for the development of articular process asymmetry. The size, shape and angulation of articular surfaces does vary across vertebral levels, but there are usually negligible left-right differences in articular process morphology within a single vertebra [20]. The articular surface area of the articular processes progressively increases from caudal C2 (~12 cm^2^) to cranial C7 (~ 20 cm^2^), but then undergoes a substantial reduction in surface area from caudal C7 (~15 cm^2^) to caudal T2 (~ 5 cm^2^), where the articular surface areas to remain fairly consistent through the remaining cranial thoracic region (unpublished data; KK Haussler). The reduction in articular surface area from C7-T2, combined with the abrupt transition from a freely moveable cervical region to a relative fixed cranial thoracic region, may increase local stresses that could contribute to the higher prevalence of osseous changes found in this region [40].

The stages of articular margin lipping and modeling found in our study readily demonstrate a progressive increase in size and severity of bony changes. These bony proliferations differ from osteophytes by the presence of smooth, well-defined joint margins; however, the formation of lipping at joint margins is likely produced by the common pathway of endochondral ossification and progressive overgrowth of cartilage and subchondral bone [45]. The progression of joint margin lipping involves the initial development of a thin horizontal projection that develops into a dorsally- or ventrally-deviated joint margin. Continued growth and modeling result in the formation of a large overhanging joint margin with well-defined cortical and trabecular bone. The increasing size of the lipping or overhanging joint margin induces moderate to severe flattening or regressive modeling of the opposing articular surface. The opposite scenario would be that in which flattening or articular surface deflection is the inciting factor for secondary overgrowth of the opposing articular joint margins. This situation seems unlikely as there is no impetus for the flattening to develop without a primary impact or influence by overlapping bony proliferation.

Bone remodeling and modeling are commonly used terms but are often misconstrued in reference to the bony proliferation associated with osteoarthritis. The adult skeleton is constantly renewed by bone remodeling, which is the process by which osteoclasts and osteoblasts work sequentially within the same bone remodeling unit to repair or replace bone via balanced resorption and subsequent bone matrix formation [47, 48]. In contrast, bone modeling describes the process whereby bones are shaped or reshaped by the independent action of osteoblast and osteoclasts. Bone modeling in osteoarthritis occurs primarily at the subchondral bone plate by either increased thickness and volume (i.e., progressive modeling) or by retraction of the subchondral bone surface (i.e., regressive modeling or bone attrition) [49]. Progressive modeling (i.e., increased formation) is the process by which the subchondral bone mass moves forward and the tidemark is thickened due to increased cartilage calcification. Progressive modeling could be seen externally as an enlargement in the overall bone shape as was noted in severe overhanging joint margins that had normal-appearing cortical bone thickness and underlying trabecular bone architecture on transverse sections.

Regressive modeling could be seen externally in our study as induced flattening or angular deviation in the articular surface [50]. It is possible that the flattening or articular surface deflection is a congenital condition and not developmental, as moderate flattening without notable osteophytic changes was noted in a young (i.e., 2-year-old) horse. Impingement of the caudal joint margin of the caudal articular processes was most noticeable during induced extension of the affected vertebral segments. The flattened or deviated articular surface of the caudal articular process is covered with hyaline cartilage and is continuous with the normal articular surface. The site of impingement or impact on the adjacent dorsal vertebral lamina is covered in fibrocartilage and is contiguous with the normal hyaline-covered articular surface (unpublished data; KK Haussler).

It is unknown how articular process thickening (i.e., increased distance between the joint capsule insertion and the joint margin) develops as the joint capsule attachments and periarticular bone within the joint capsule margins at affected sites appeared grossly normal. One hypothesis is that the articular process supporting the articular surface thickened via bone modeling which resulted in the joint margin moving away from the joint capsule insertion site. Within an opposing pair of articular surfaces, changes in one articular process causes an opposite response in its counterpart, such that if one surface undergoes regressive bone modeling characterized by resorption of subchondral bone support, then the opposing articular surface undergoes progressive bone modeling in an attempt to follow and encompass the loss and shape of surface area by its counterpart [51]. An alternative hypothesis is that the joint capsule insertion migrated away from the joint margin, resulting in the increased distance or the appearance of articular process thickening. During bone growth and modeling, tendon and ligament attachments do maintain a proportional distance from the articular surfaces with bone elongation associated with epiphyseal expansion [52]. As the periosteum contributes to bone formation on the cortical surfaces and is contiguous with enthesis, it has been hypothesized that the periosteum plays a role in migration of tendon and ligament attachments via both osteogenic and resorptive processes [53]. In human knees, the distance from the tibial tuberosity to the joint margin is quite variable [54]. The complex interphase at the soft tissue attachment to bone that functions in stress dissipation during mechanical loading and exercise has been termed the ‘enthesis organ’ [55]. Sources of joint capsule stress include mechanical stretching due to joint effusion or during end ranges of joint motion [56]. As there was no ultrasonographic evaluation of the affected articulations, it is unknown if there was a correlation with joint effusion or what effects that chronic joint capsule distension might have on enthesis development or possible migration. A final hypothesis is that this is a congenital variant of normal joint capsule attachment on the articular process. No matter the underlying etiology, it appears that the affected joint capsule would be enlarged in these affected synovial articulations. The clinical relevance of this unique osseous change remains unknown.

Soft tissue inflammation associated with chronic osteoarthritis has been reported to produce osteolytic lesions where resorptive processes can localize or outweigh bony proliferation during osteophyte formation [57]. During bone development, epiphyseal arteries that arise from a periarticular vascular plexus near the ends of long bones enter the epiphysis via vascular channels [58]. During periods of excessive bone proliferation associated with osteophyte formation, there is a concurrent increase in blood flow and perivascular osteoclastic activation [59]. The resultant enlarged vascular channels often appear as and can be mistaken for periarticular lytic lesions.

The pathophysiology of enthesophyte formation can occur via several proposed mechanisms. Endochondral ossification of enthesis fibrocartilage can lead to enthesophyte formation at joint capsule, ligament or tendon insertion sites [60]. Additional mechanisms include microtears of the enthesis due to repetitive strain associated with microtrauma or joint instability that leads to fibrocyte proliferation, granulomatous formation, and eventual ossification. Enthesophytes can also form due to the production of reactive bone at an enthesis from abnormal mechanical stresses applied to the soft tissue-bony interface [45]. The identification of periosteal new bone formation in our study was likely evidence of reactive bone formation at joint capsule, ligament and muscle insertion sites, which was in the process of eventual enthesophyte formation.

Enthesophytes tend to occur in characteristic locations associated with sites of joint instability due to increased stain at adjacent muscle, tendon or ligament attachment sites. In our study, enthesophytes did occur in consistent locations that were associated with insertion sites of the multifidi and intertransversarius muscles. Joint capsule enthesophytes initially appeared at a small site locally and progressed to a circumferential distribution in severe cases. Entheses usually have an abundant nerve supply that includes proprioceptive and nociceptive receptors [61]. Therefore, pain and soft tissue reactions associated with enthesophyte formation can cause reduced joint range of motion and produce a condition of constant static loading, which can propagate local mechanobiological mechanisms associated with continued enthesophyte formation [45].

### Limitations

Due to the lack of complete medical or performance histories, the clinical significance for the described lesions is unknown; however, osteoarthritis and osteophytes at other spinal regions have been associated with axial skeleton pain and poor performance [62]. We only assessed osseous changes within these prepared specimens and were unable to assess soft tissue lesions such as cartilage loss, synovitis or joint capsule fibrosis. It is likely that gross and histological evaluation of associated osseous and soft tissues would have provided greater insights into the pathogenesis and clinical relevance of these lesions. We chose to not include findings from the occiput-C1-C2 articulations as they lack paired articular processes. Future work will need to assess bony changes in these highly mobile vertebral segments, especially in horses trained with extreme head and neck postures that repetitively exceed normal joint ranges of motion which would be expected to produce increase local stresses and cause an increased prevalence of osseous pathology [63].

## Conclusions

A large percentage of articular processes had bony changes that were considered abnormal. A wide variety of types of osseous changes were identified in both cranial and caudal articular processes. Osteophytes, lipping, joint capsule entheses and articular process thickening all were more severe within the cranial articular processes; whereas, the caudal articular process had more severe flattening and modeling of the joint margins due likely to differences in anatomical and biomechanical influences. Overall grades of severity were mostly mild to moderate with mild changes localized to C3-C6, moderate changes at C6-T2 and severe changes noted at C2-C3 and C6-T2. The more severe changes at C2-C3 may be due to induced head and neck postures; whereas, the severe changes at C6-T2 likely reflect increased local stresses due the transition from a freely moveable cervical region to a more rigid cranial thoracic region. The grade of osseous pathology was positively associated with both age and wither height due to larger horses being predisposed to increased local stresses associated with longer and heavier head and necks.

We attempted to identify a progression in the type and severity of osseous lesions to better understand the pathogenesis of cervical osteoarthritis and associated changes in bone morphology. The results of this study provide information on the prevalence, vertebral distribution, and articular location of osseous changes which are expected to increase awareness for future clinical and diagnostic imaging studies. Unfortunately, we were not able to provide any measure of clinical relevance of the reported findings.

The authors believe that more descriptive pathologic and radiographic terms other than “osteophytes” are needed to fully capture the spectrum of disease localized to articular processes. Unfortunately, there are a wide variety of confusing and poorly defined pathologic and radiographic terms used to describe joint disease within the cervical region of horses [8, 24]. There is a need to develop standardized descriptions and terminology of abnormal gross and radiographic findings to better characterize and assess the wide spectrum of osseous and soft tissue changes within the cervical region, especially with the advent of advanced imaging technology. It is hoped that some of the descriptors and terminology used in this pathoanatomical study will contribute to that need.

## Supporting information

S1 Table Prevalence of grades of severity of the different types of bony changes within cranial and caudal articular processes across all subjects. (DOCX)

S2 Table Prevalence of bony changes within each articular quadrant of the cranial articular processes. (DOCX)

S3 Table Prevalence of bony changes within each articular quadrant of the caudal articular processes. (DOCX)

S4 Table Prevalence of bony changes within each vertebral level (C2-T3) for the cranial articular processes. (DOCX)

S5 Table Prevalence of bony changes within each vertebral level (C2-T3) for the caudal articular processes. (DOCX)

S6 Table Grades of severity of bony changes within paired caudal (Cd)-Cranial (Cr) articular processes within a single synovial articulation. (DOCX)

S7 Table Grades of severity of bony changes between Left (L)-Right (R) paired articular processes. (DOCX)

S8 Table Grades of severity of bony changes within cranial (Cr) versus caudal (Cd) articular processes within a vertebra. (DOCX)

S1 Fig STROBE (strengthening the reporting of observational studies in epidemiology) checklist. (PDF)
