# Supplementary material for: Characterization of bony changes localized to the cervical articular processes in a mixed population of horses
Source: PLoS One. 2019 Sep 26;14(9):e0222989. doi: 10.1371/journal.pone.0222989 (PMC6762202; doi:10.1371/journal.pone.0222989)
Supplement: S1 Table — (DOCX) [file pone.0222989.s001.docx]

|  | **Cranial Articular Processes** | | | **Caudal Articular Processes** | | |
| --- | --- | --- | --- | --- | --- | --- |
| **Osseous changes** | **Mild** | **Moderate** | **Severe** | **Mild** | **Moderate** | **Severe** |
| Osteophyte | 18% | 11% | 2.7% | 8% | 8% | 1.3% |
| Flattening | 7% | 2% | 0.1% | 25% | 11% | 0.1% |
| Lipping | 25% | 3% | 0.0% | 17% | 2% | 0.1% |
| Modeling | 1% | 0.2% | 0.0% | 14% | 6% | 0.6% |
| Joint capsule enthesis | 5% | 5% | 0.6% | 0.3% | 1% | 0.2% |
| Thickening | 4% | 2% | 0.0% | 0.2% | 1% | 0.1% |
| Extension impingement | 2% | 4% | 0.6% | 2% | 1% | 0.0% |
| Enlarged vascular channels | 1% | 2% | 0.5% | 1% | 1% | 0.1% |
| Muscle enthesis | 1% | 1% | 0.2% | 1% | 2% | 0.2% |
| Asymmetry | 0.8% | 0.1% | 0.0% | 0.9% | 0.0% | 0.1% |
| Periosteal callus | 0.2% | 0.5% | 0.2% | 0.1% | 0.2% | 0.0% |
| Ankylosis | 0.0% | 0.0% | 0.2% | 0.0% | 0.0% | 0.2% |
