# Supplementary material for: Characterization of bony changes localized to the cervical articular processes in a mixed population of horses
Source: PLoS One. 2019 Sep 26;14(9):e0222989. doi: 10.1371/journal.pone.0222989 (PMC6762202; doi:10.1371/journal.pone.0222989)
Supplement: S2 Table — (DOCX) [file pone.0222989.s002.docx]

|  | **Articular quadrant** | | | | |
| --- | --- | --- | --- | --- | --- |
| **Osseous changes** | **Cranial** | **Caudal** | **Medial** | **Lateral** | **Periarticular** |
| Osteophyte | 44% | 8% | 21% | 19% | 25% |
| Flattening | 26% | 0% | 19% | 55% | 0% |
| Lipping | 13% | 4% | 60% | 19% | 4% |
| Modeling | 14% | 0% | 29% | 57% | 0% |
| Joint capsule enthesis | 0% | 37% | 0% | 59% | 4% |
| Thickening | 2% | 0% | 0% | 0% | 98% |
| Extension impingement | 2% | 90% | 8% | 0% | 0% |
| Enlarged vascular channels | 6% | 39% | 21% | 6% | 27% |
| Intertransverse muscle enthesis | 7% | 7% | 0% | 87% | 0% |
| Asymmetry | 0% | 0% | 0% | 0% | 100% |
| Periosteal callus | 0% | 33% | 67% | 0% | 0% |
| Ankylosis | 0% | 0% | 0% | 0% | 100% |
| **Pooled** | 21% | 16% | 27% | 19% | 17% |
