# Supplementary material for: Characterization of bony changes localized to the cervical articular processes in a mixed population of horses
Source: PLoS One. 2019 Sep 26;14(9):e0222989. doi: 10.1371/journal.pone.0222989 (PMC6762202; doi:10.1371/journal.pone.0222989)
Supplement: S3 Table — (DOCX) [file pone.0222989.s003.docx]

|  | **Articular quadrant** | | | | |
| --- | --- | --- | --- | --- | --- |
| **Osseous changes** | **Cranial** | **Caudal** | **Medial** | **Lateral** | **Periarticular** |
| Osteophyte | 5% | 31% | 23% | 8% | 34% |
| Flattening | 0% | 13% | 87% | 0% | 0% |
| Lipping | 6% | 6% | 26% | 53% | 9% |
| Modeling | 0% | 1% | 0% | 98% | 1% |
| Joint capsule enthesis | 10% | 20% | 0% | 60% | 10% |
| Thickening | 0% | 0% | 0% | 0% | 100% |
| Extension impingement | 0% | 30% | 70% | 0% | 0% |
| Enlarged vascular channels | 27% | 7% | 27% | 0% | 40% |
| Multifidi muscle enthesis | 0% | 0% | 0% | 100% (dorsal) | 0% |
| Asymmetry | 0% | 0% | 0% | 0% | 100% |
| Periosteal callus | 0% | 0% | 0% | 67% | 33% |
| Ankylosis | 0% | 0% | 0% | 0% | 100% |
| **Pooled** | 2% | 12% | 42% | 34% | 10% |
