# Supplementary material for: Characterization of bony changes localized to the cervical articular processes in a mixed population of horses
Source: PLoS One. 2019 Sep 26;14(9):e0222989. doi: 10.1371/journal.pone.0222989 (PMC6762202; doi:10.1371/journal.pone.0222989)
Supplement: S4 Table — (DOCX) [file pone.0222989.s004.docx]

|  | **Vertebral level** | | | | | | | |
| --- | --- | --- | --- | --- | --- | --- | --- | --- |
| **Osseous changes** | **C3** | **C4** | **C5** | **C6** | **C7** | **T1** | **T2** | **T3** |
| Osteophyte | 47% | 15% | 19% | 20% | 39% | 48% | 45% | 16% |
| Flattening | 10% | 19% | 11% | 6% | 15% | 7% | 2% | 0% |
| Lipping | 15% | 53% | 50% | 41% | 12% | 13% | 14% | 20% |
| Modeling | 5% | 0% | 1% | 1% | 0% | 0% | 0% | 0% |
| Joint capsule enthesis | 9% | 23% | 25% | 20% | 8% | 1% | 0% | 1% |
| Thickening | 1% | 15% | 15% | 3% | 5% | 6% | 1% | 0% |
| Extension impingement | 2% | 0% | 0% | 2% | 3% | 49% | 2% | 0% |
| Enlarged vascular channels | 0% | 0% | 2% | 4% | 5% | 13% | 3% | 1% |
| Intertransverse muscle enthesis | 4% | 6% | 3% | 0% | 1% | 0% | 0% | 0% |
| Asymmetry | 0% | 0% | 0% | 1% | 0% | 1% | 1% | 4% |
| Periosteal callus | 1% | 0% | 0% | 4% | 1% | 2% | 0% | 0% |
| Ankylosis | 2% | 0% | 0% | 0% | 0% | 0% | 0% | 0% |
