# Supplementary material for: Characterization of bony changes localized to the cervical articular processes in a mixed population of horses
Source: PLoS One. 2019 Sep 26;14(9):e0222989. doi: 10.1371/journal.pone.0222989 (PMC6762202; doi:10.1371/journal.pone.0222989)
Supplement: S5 Table — (DOCX) [file pone.0222989.s005.docx]

|  | **Vertebral level** | | | | | | | | | |  |
| --- | --- | --- | --- | --- | --- | --- | --- | --- | --- | --- | --- |
| **Osseous changes** | | **C2** | **C3** | **C4** | **C5** | **C6** | **C7** | **T1** | **T2** | **T3** | |
| Osteophyte | | 12**%** | 8% | 15% | 12% | 21% | 42% | 19% | 18% | 11% | |
| Flattening | | 41% | 22% | 32% | 45% | 54% | 65% | 66% | 3% | 1% | |
| Lipping | | 24% | 24% | 44% | 18% | 19% | 8% | 17% | 17% | 7% | |
| Modeling | | 27% | 42% | 32% | 15% | 29% | 15% | 17% | 4% | 0% | |
| Joint capsule enthesis | | 1% | 0% | 0% | 2% | 1% | 4% | 2% | 0% | 0% | |
| Thickening | | 0% | 0% | 0% | 2% | 5% | 1% | 0% | 0% | 0% | |
| Extension impingement | | 1% | 0% | 0% | 2% | 6% | 5% | 6% | 0% | 1% | |
| Enlarged vascular channels | | 0% | 1% | 0% | 5% | 4% | 3% | 1% | 0% | 0% | |
| Multifidi muscle enthesis | | 0% | 0% | 5% | 5% | 8% | 10% | 0% | 0% | 0% | |
| Asymmetry | | 0% | 1% | 0% | 0% | 0% | 0% | 0% | 5% | 4% | |
| Periosteal callus | | 0% | 0% | 0% | 0% | 0% | 0% | 0% | 0% | 3% | |
| Ankylosis | | 2% | 0% | 0% | 0% | 0% | 0% | 0% | 0% | 0% | |
