# Supplementary material for: Characterization of bony changes localized to the cervical articular processes in a mixed population of horses
Source: PLoS One. 2019 Sep 26;14(9):e0222989. doi: 10.1371/journal.pone.0222989 (PMC6762202; doi:10.1371/journal.pone.0222989)
Supplement: S6 Table — (DOCX) [file pone.0222989.s006.docx]

| **Cd-Cr Pairs** | **Cd-Cr Same** | **Cd > Cr** | **Cr > Cd** | **Signed Rank** |
| --- | --- | --- | --- | --- |
| C2-C3 Left AP | 47% | 19% | 34% | P = 0.110 |
| C2-C3 Right AP | 48% | 24% | 28% | P = 0.646 |
| C3-C4 Left AP | 51% | 35% | 15% | P = 0.106 |
| C3-C4 Right AP | 47% | 18% | 35% | P = 0.093 |
| C4-C5 Left AP | 64% | 15% | 22% | P = 0.621 |
| C4-C5 Right AP | 51% | 24% | 25% | P = 0.976 |
| C5-C6 Left AP | 43% | 24% | 33% | P = 0.310 |
| C5-C6 Right AP | 54% | 24% | 22% | P = 0.864 |
| C6-C7 Left AP | 50% | 39% | 11% | P = 0.062 |
| C6-C7 Right AP | 33% | 57% | 9% | P = 0.001 |
| C7-T1 Left AP | 49% | 29% | 22% | P = 0.364 |
| C7-T1 Right AP | 45% | 25% | 29% | P = 0.953 |
| T1-T2 Left AP | 41% | 41% | 19% | P = 0.035 |
| T1-T2 Right AP | 28% | 54% | 19% | P = 0.003 |
| T2-T3 Left AP | 64% | 26% | 10% | P = 0.078 |
| T2-T3 Right AP | 60% | 22% | 18% | P = 0.482 |
| **Pooled** | 52% | 28% | 20% |  |
