# Supplementary material for: Characterization of bony changes localized to the cervical articular processes in a mixed population of horses
Source: PLoS One. 2019 Sep 26;14(9):e0222989. doi: 10.1371/journal.pone.0222989 (PMC6762202; doi:10.1371/journal.pone.0222989)
Supplement: S7 Table — (DOCX) [file pone.0222989.s007.docx]

| **L-R Vertebral Level** | **L-R Same** | **L > R** | **R > L** | **Signed Rank** |
| --- | --- | --- | --- | --- |
| C2 Caudal AP | 60% | 18% | 22% | P = 0.674 |
| C3 Cranial AP | 53% | 29% | 18% | P = 0.293 |
| C3 Caudal AP | 44% | 44% | 13% | P = 0.002 |
| C4 Cranial AP | 67% | 16% | 16% | P = 0.849 |
| C4 Caudal AP | 64% | 20% | 16% | P = 0.699 |
| C5 Cranial AP | 73% | 16% | 11% | P = 0.279 |
| C5 Caudal AP | 64% | 15% | 22% | P = 0.532 |
| C6 Cranial AP | 60% | 20% | 20% | P = 0.776 |
| C6 Caudal AP | 53% | 16% | 31% | P = 0.105 |
| C7 Cranial AP | 60% | 24% | 16% | P = 0.374 |
| C7 Caudal AP | 64% | 16% | 20% | P = 1.000 |
| T1 Cranial AP | 67% | 11% | 22% | P = 0.224 |
| T1 Caudal AP | 63% | 9% | 28% | P = 0.049 |
| T2 Cranial AP | 78% | 9% | 13% | P = 0.371 |
| T2 Caudal AP | 70% | 9% | 13% | P = 0.204 |
| T3 Cranial AP | 77% | 4% | 19% | P = 0.030 |
| T3 Caudal AP | 67% | 13% | 19% | P = 0.627 |
| **Pooled** | 68% | 15% | 17% |  |
