# Supplementary material for: Characterization of bony changes localized to the cervical articular processes in a mixed population of horses
Source: PLoS One. 2019 Sep 26;14(9):e0222989. doi: 10.1371/journal.pone.0222989 (PMC6762202; doi:10.1371/journal.pone.0222989)
Supplement: S8 Table — (DOCX) [file pone.0222989.s008.docx]

| **Cr-Cd Sites** | **Cr-Cd Same** | **Cr > Cd** | **Cd > Cr** | **Signed Rank** |
| --- | --- | --- | --- | --- |
| C3 Left Cr-Cd AP | 43% | 22% | 34% | P = 0.348 |
| C3 Right Cr-Cd AP | 43% | 33% | 24% | P = 0.371 |
| C4 Left Cr-Cd AP | 60% | 21% | 19% | P = 0.871 |
| C4 Right Cr-Cd AP | 64% | 18% | 18% | P = 0.737 |
| C5 Left Cr-Cd AP | 60% | 30% | 10% | P = 0.028 |
| C5 Right Cr-Cd AP | 61% | 21% | 18% | P = 0.427 |
| C6 Left Cr-Cd AP | 48% | 18% | 34% | P = 0.068 |
| C6 Right Cr-Cd AP | 52% | 10% | 37% | P = 0.003 |
| C7 Left Cr-Cd AP | 54% | 7% | 39% | P = 0.000 |
| C7 Right Cr-Cd AP | 45% | 9% | 46% | P = 0.000 |
| T1 Left Cr-Cd AP | 49% | 36% | 15% | P = 0.057 |
| T1 Right Cr-Cd AP | 49% | 33% | 18% | P = 0.173 |
| T2 Left Cr-Cd AP | 61% | 30% | 9% | P = 0.012 |
| T2 Right Cr-Cd AP | 58% | 29% | 14% | P = 0.076 |
| T3 Left Cr-Cd AP | 70% | 19% | 10% | P = 0.417 |
| T3 Right Cr-Cd AP | 69% | 22% | 9% | P = 0.059 |
| **Pooled** | 55% | 22% | 22% |  |
